# Benzylic Dehydroxylation of Echinocandin Antifungal Drugs Restores Efficacy against Resistance Conferred by Mutated Glucan Synthase

**DOI:** 10.1021/jacs.2c00269

**Published:** 2022-03-29

**Authors:** Dana Logviniuk, Qais Z. Jaber, Roman Dobrovetsky, Noga Kozer, Ewa Ksiezopolska, Toni Gabaldón, Shmuel Carmeli, Micha Fridman

**Affiliations:** † School of Chemistry, Raymond & Beverly Sackler Faculty of Exact Sciences, Tel Aviv University, Tel Aviv 6997801, Israel; ‡ The Wohl Drug Discovery institute of the Nancy and Stephen Grand Israel National Center for Personalized Medicine, Weizmann Institute of Science, Rehovot 7610001, Israel; § Barcelona Supercomputing Centre (BSC-CNS), Jordi Girona, 29, Barcelona 08034, Spain; ∥ Institute for Research in Biomedicine (IRB Barcelona), The Barcelona Institute of Science and Technology, Baldiri Reixac, 10, Barcelona 08028, Spain; ⊥ Catalan Institution for Research and Advanced Studies (ICREA), Passeig de Lluís Companys, 23, Barcelona 08010, Spain; # Centro Investigación Biomédica En Red de Enfermedades Infecciosas, Madrid 28029, Spain

## Abstract

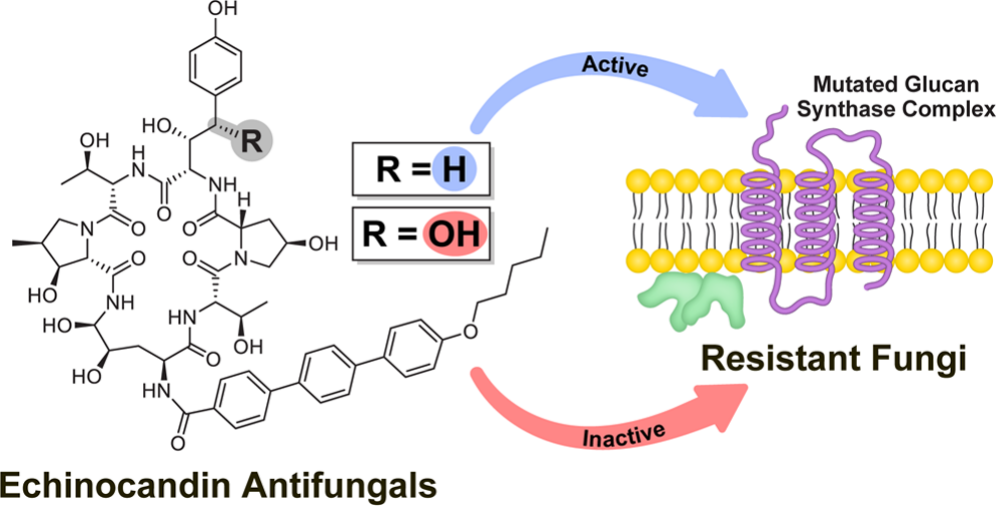

Each year, infections caused by fungal pathogens claim the lives of about 1.6 million people and affect the health of over a billion people worldwide. Among the most recently developed antifungal drugs are the echinocandins, which noncompetitively inhibit β-glucan synthase, a membrane-bound protein complex that catalyzes the formation of the main polysaccharide component of the fungal cell wall. Resistance to echinocandins is conferred by mutations in *FKS* genes, which encode the catalytic subunit of the β-glucan synthase complex. Here, we report that selective removal of the benzylic alcohol of the nonproteinogenic amino acid 3*S*,4*S*-dihydroxy-l-homotyrosine of the echinocandins anidulafungin and rezafungin, restored their efficacy against a large panel of echinocandin-resistant *Candida* strains. The dehydroxylated compounds did not significantly affect the viability of human-derived cell culture lines. An analysis of the efficacy of the dehydroxylated echinocandins against resistant *Candida* strains, which contain mutations in the *FKS*1 and/or *FKS*2 genes of the parental strains, identified amino acids of the Fks proteins that are likely to reside in proximity to the l-homotyrosine residue of the bound drug. This study describes the first example of a chemical modification strategy to restore the efficacy of echinocandin drugs, which have a critical place in the arsenal of antifungal drugs, against resistant fungal pathogens.

## Introduction

It is estimated that approximately 13% of the world’s population suffers from fungal diseases each year worldwide and that fungal infections cause about 1.6 million annual fatalities.^1^ The increasing prevalence of drug-resistant (and sometimes multidrug-resistant) fungal pathogens, including *Aspergillus fumigatus*, *Candida glabrata*, *Cryptococcus neoformans*, and *Candida auris*, the latter is a pathogen with the potential for extensive multidrug resistance, poses a major health challenge, especially in light of the paucity of antifungal drug classes.^2−5^ The human genome shares high similarities with that of pathogenic fungi, which may explain why, compared to the relative abundance of drug targets in bacteria, fewer targets exist in fungi and very few classes of antifungal drugs have been developed to date.^6−10^

**Figure 1:**
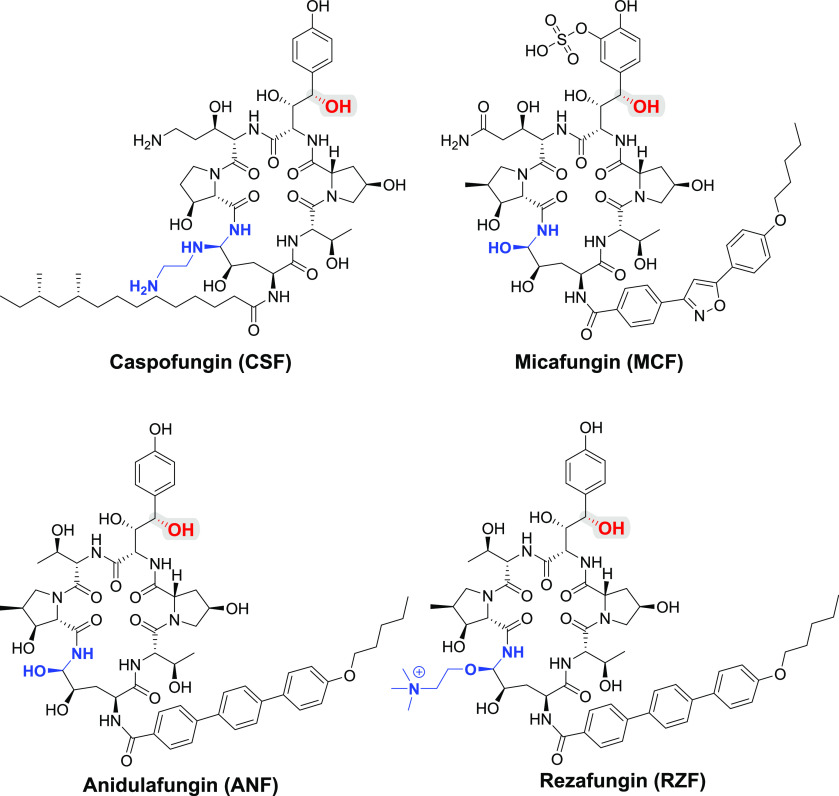
Structures of the four echinocandins in clinical use or in clinical trials. The echinocandin drug scaffold is colored black. The aminal, hemiaminal, and choline-based hemiaminal ether functionalities are colored in blue. The benzylic position is highlighted by red-colored atoms and gray background.

Clinically used antifungal drugs belong to four major drug classes: azoles, allylamines, polyenes, and echinocandins.^4,11−13^ In the approximately 20 years since the approval of the first of the echinocandins, the most recently developed of the four classes, these have become the drugs of choice for treating candidemia and invasive candidiasis,^14^ severe life-threatening infections caused by pathogenic yeast of the genus *Candida*, the most prevalent fungal pathogen in humans.^15^ Currently, this class of antifungals includes only three drugs approved for clinical use (Figure 1): caspofungin (CSF), micafungin (MCF), and anidulafungin (ANF); a fourth, rezafungin (RZF), is currently undergoing phase III clinical testing.^11^

Echinocandins interfere with the biosynthesis of the fungal cell wall through noncompetitive inhibition of β-(1 → 3)-glucan synthase (GS), a membrane-bound complex that polymerizes the main fungal cell-wall polysaccharide component.^16^ The GS complex has at least two subunits, Fks and Rho. The Fks subunit catalyzes the transfer of sugar moieties from an activated glucosyl donor (UDP-glucose) to the growing glucan polysaccharide.^17^ Fks proteins are encoded by three related genes, *FKS*1, *FKS*2, and *FKS*3.^17^ Echinocandin resistance is associated with point mutations in “hot spot” (HS) regions of the essential *FKS* genes.^18−27^ In *Candida albicans*, two additional *FKS* genes, *FKS*2 and *FKS*3, have high sequence similarity to *FKS*1, yet evidence indicates that these genes encode proteins that have regulatory roles.^28^ In *C. glabrata*, *FKS*1 and *FKS*2 are functionally redundant, and echinocandin resistance can result from mutations in either gene, although they are more frequently encountered in *FKS*2.^27,29^

The evolution of resistance is the ultimate fate of all antimicrobial drugs, and echinocandins are no exception. In recent years, the prevalence of echinocandin resistance in clinical isolates has increased.^30−37^ Just 4 years after the FDA approved the first echinocandin, the first mutations in the *FKS* genes that confer resistance to echinocandins were discovered.^38^ These mutations reduce the affinity of the target Fks protein for the drug, resulting in higher minimum inhibitory concentration (MIC) values.^39^ During the last decade, *C. glabrata* has become one of the most frequent causes of severe fungal infections in the United States. This *Candida* species has acquired resistance to many of the antifungal drugs from the azole class,^40^ and during the first decade after the approval of CSF for clinical use, the incidence of echinocandin resistance in *C. glabrata* increased from 4.9 to 12%.^41^ In addition to the rapid increase in echinocandin resistance, pharmacokinetic properties, including a high affinity for blood plasma proteins, rapid elimination by hepatic metabolism, and spontaneous degradation limit the use of echinocandins.^42^ Furthermore, due to the limited absorption of oral formulations, all of the currently used echinocandins are administered daily intravenously.^43^

High-resolution structures of the target Fks protein alone or in complex with an echinocandin are not yet available. In the absence of structures to guide modification of the drugs to improve affinity for Fks, the development of next-generation echinocandins has focused on improving pharmacokinetic and pharmacodynamic properties. With the goal of developing echinocandins with improved stability under physiological conditions, Cidara Therapeutics developed RZF, which is generated from ANF via a single-step conversion of the hemiaminal of ANF to a choline-based hemiaminal ether that is more hydrolytically stable than the hemiaminal in ANF (Figure 1). As a result of improved stability, instead of a daily intravenous administration, RZF can be administered intravenously once a week.^44,45^

Single-atom or single-functional group changes in bioactive molecules can lead to profound effects on their activity.^46−54^ Even the smallest chemical modification can change steric, electronic, and conformational properties or hydrogen bonding and alter the set of interactions between the molecule and the target. For example, hydroxyl groups are critical for activities of antibiotics of different classes: Through the total synthesis of a derivative of the last-resort antifungal polyene drug amphotericin B, Carreira and co-workers demonstrated the importance of the hydroxyl group at C35, one of 10 alcohol groups in this drug, and the involvement of double-barrel ion channels in its mechanism of action.^49^ Miller and co-workers used site-selective modifications including thiocarbonylation and deoxygenation of the glycopeptide antibiotic vancomycin to show that hydroxyl groups stabilize the active conformation of this antibiotic.^51^ To overcome resistance caused by enzymatic deactivation, the aminoglycoside antibiotic dibekacin was developed from kanamycin B by removal of the 3-OH and 4-OH of the parent aminoglycoside antibiotic.^52^

We postulated that this strategy could be implemented to enhance the affinity of echinocandins for the mutated Fks expressed in resistant isolates. In search of a chemical modification site that might restore the efficacy of echinocandins against resistant fungi, we reviewed their biosynthetic pathway. Echinocandin biosynthesis was first investigated by researchers from Merck & Co. using ^13^C-labeling experiments carried out with the pneumocandin-producer *Zalerion arboricola*.^55,56^ Tang and Walsh identified and characterized the enzymes involved in the biosynthesis of echinocandin B in *Emericella rugulosa* NRRL 11440.^57^ Their breakthrough study paved the way for the generation of mutants capable of producing novel echinocandin derivatives.^58^ Our attention was drawn to the fact that structure–activity investigation revealed that side-chain hydroxylation of l-homotyrosine is not essential for antifungal activity.^59−61^ This suggested to us that removal of the benzylic alcohol of 3*S*,4*S*-dihydroxy-l-homotyrosine could potentially reduce steric clashes between the echinocandin and amino acid residues in the mutated drug-binding pocket and lead to a reduction or possibly abrogation of resistance. To test our hypothesis, we chemoselectively removed the benzylic alcohol of 3*S*,4*S*-dihydroxy-l-homotyrosine from ANF and RZF and showed that this modification enhanced the efficacy against a large panel of echinocandin-resistant *Candida* pathogens. The data generated provide insights into the binding site of this class of antifungal drugs in the currently uncharacterized Fks catalytic subunit of the GS complex.

## Results and Discussion

### Synthesis of 3*S*-Hydroxy-l-homotyrosine Derivatives of ANF and RZF through a Single-Step, Chemoselective Hydrogenation

The cyclic hexapeptide of anidulafungin contains nine alcohol groups, including secondary alcohols, a phenol, a hemiaminal, and benzylic and homobenzylic alcohols, making it challenging to predict the outcomes of reductive dehydroxylation of this complex molecule. In 1994, researchers from Merck & Co., reported the use of triethylsilane and trifluoroacetic acid for selective removal of the benzylic alcohol of pneumocandin B_0_, the natural echinocandin precursor used for the preparation of the echinocandin drug CSF, in diethyl ether containing 2 M LiClO_4_.^62^ Due to solubility limitations of ANF, we carried out this reaction in THF and only traces of conversion were observed. In 1999, an Eli Lilly team reported on the double dehydroxylation of the benzylic alcohol and the hemiaminal of ANF using triethylsilane and trifluoroacetic acid in dichloromethane;^63^ a reaction for selective removal of the benzylic alcohol from ANF was not reported.

**Scheme 1:**
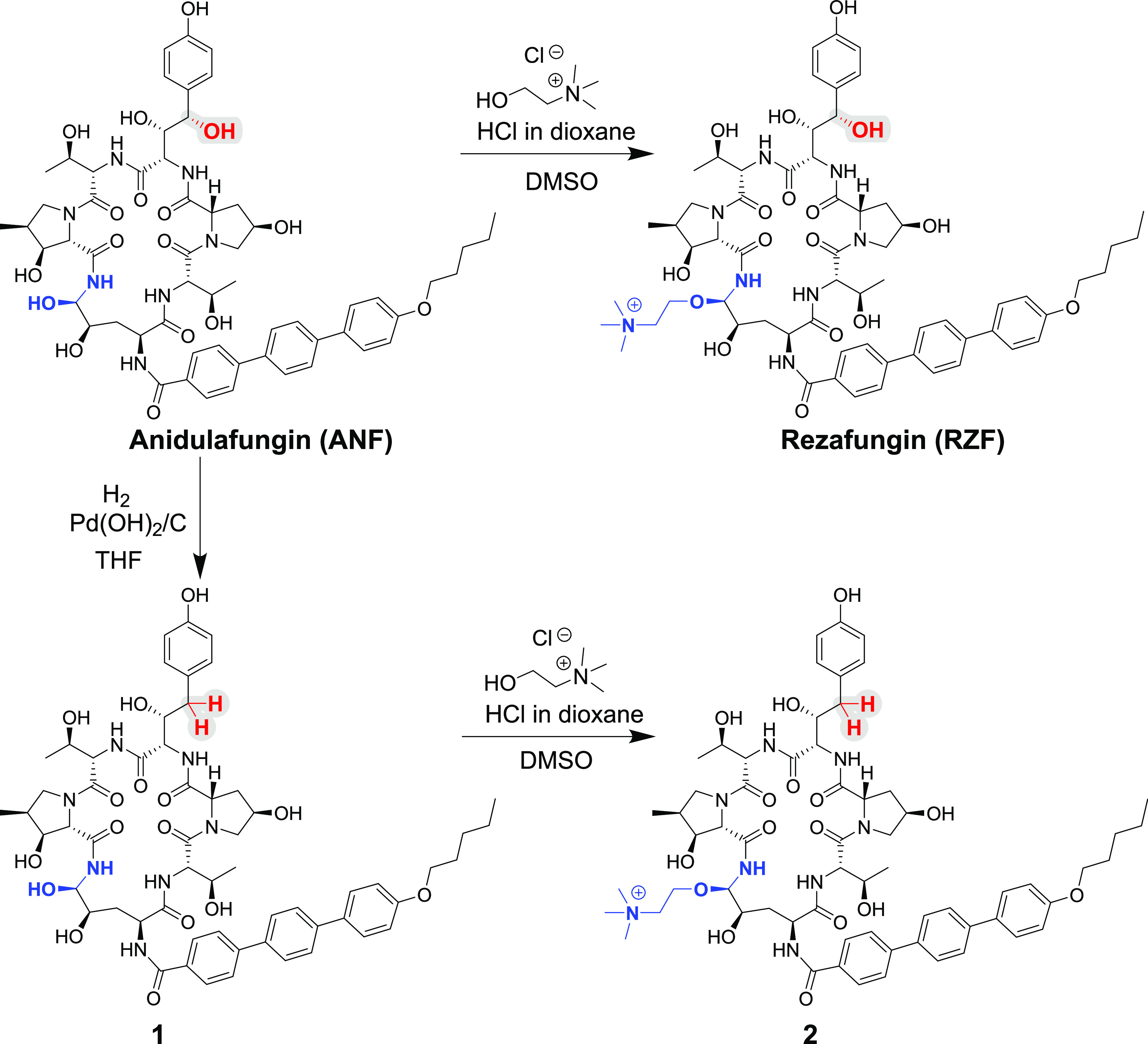
Synthesis of 3*S*-Hydroxy-l-homotyrosine Derivatives of ANF and RZF Echinocandin drug scaffold is colored black. Hemiaminal and choline hemiaminal ether functionalities of ANF and RZF, respectively, are colored in blue. Modification position is highlighted by red-colored atoms and gray background.

Treatment of ANF with Pd(OH)_2_/C and hydrogen in THF for 48 h at ambient temperature resulted in selective removal of the benzylic alcohol of ANF with approximately 50% formation of the desired product (compound **1**, Scheme 1). Of note, no benzylic dehydroxylation product was observed when the same conditions used to generate compound **1** from ANF were applied to RZF. RZF derivative **2** was formed (approximately 37% conversion) by treatment of compound **1** with excess choline chloride and dry HCl in dioxane with DMSO as a solvent (Scheme 1).

**Figure 2:**
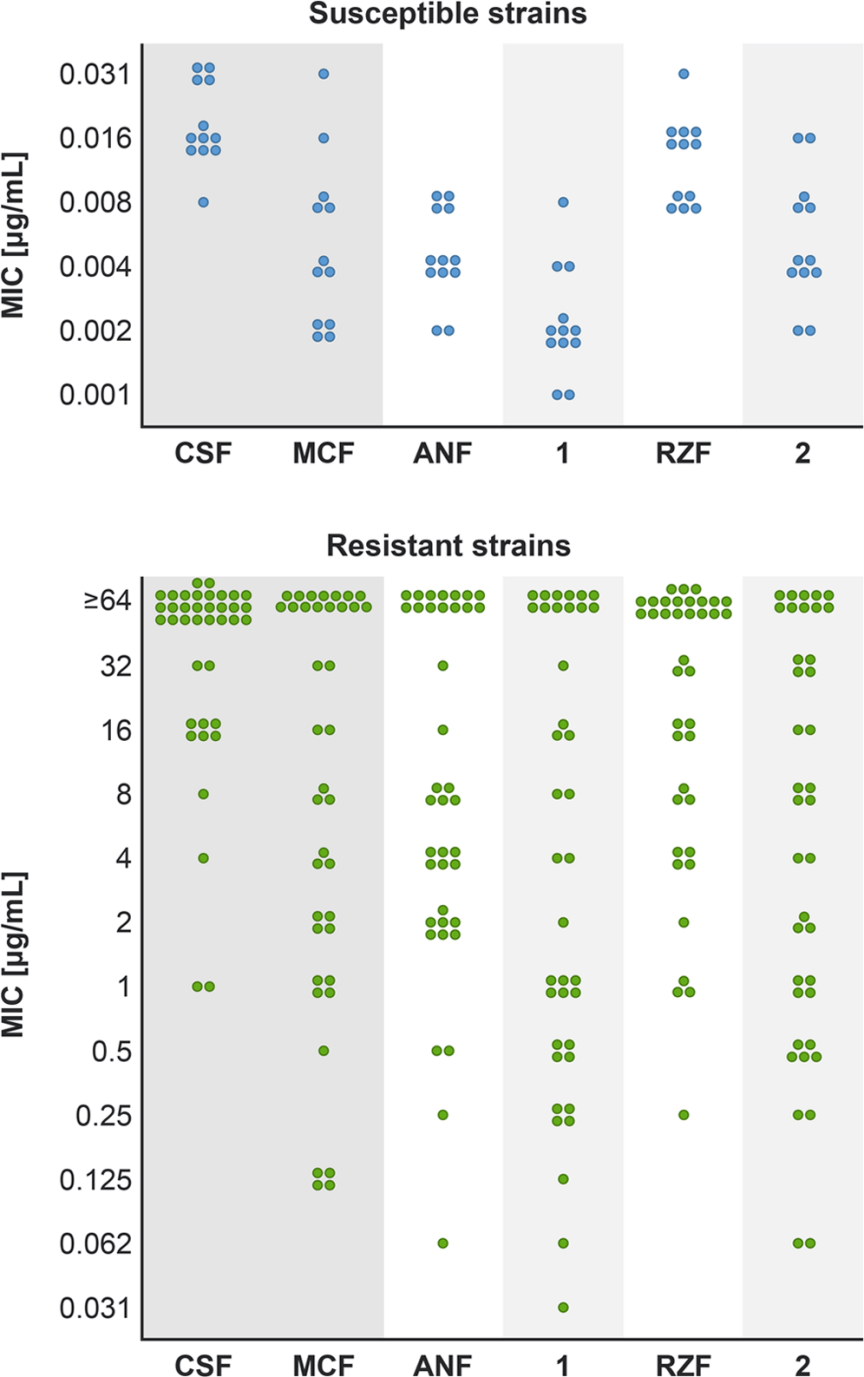
Antifungal activities of the four echinocandins in clinical use or in clinical trials (CSF, MCF ANF, and RZF) and of dehydroxylated echinocandins **1** and **2**. MIC values for echinocandin-susceptible strains are shown in the top panel and those for echinocandin-resistant strains are shown in the bottom panel. Each concentration was tested in triplicate, and results were confirmed by at least two independent sets of experiments. MIC values for each specific strain in the panel are summarized in Table S5.

**Figure 3:**
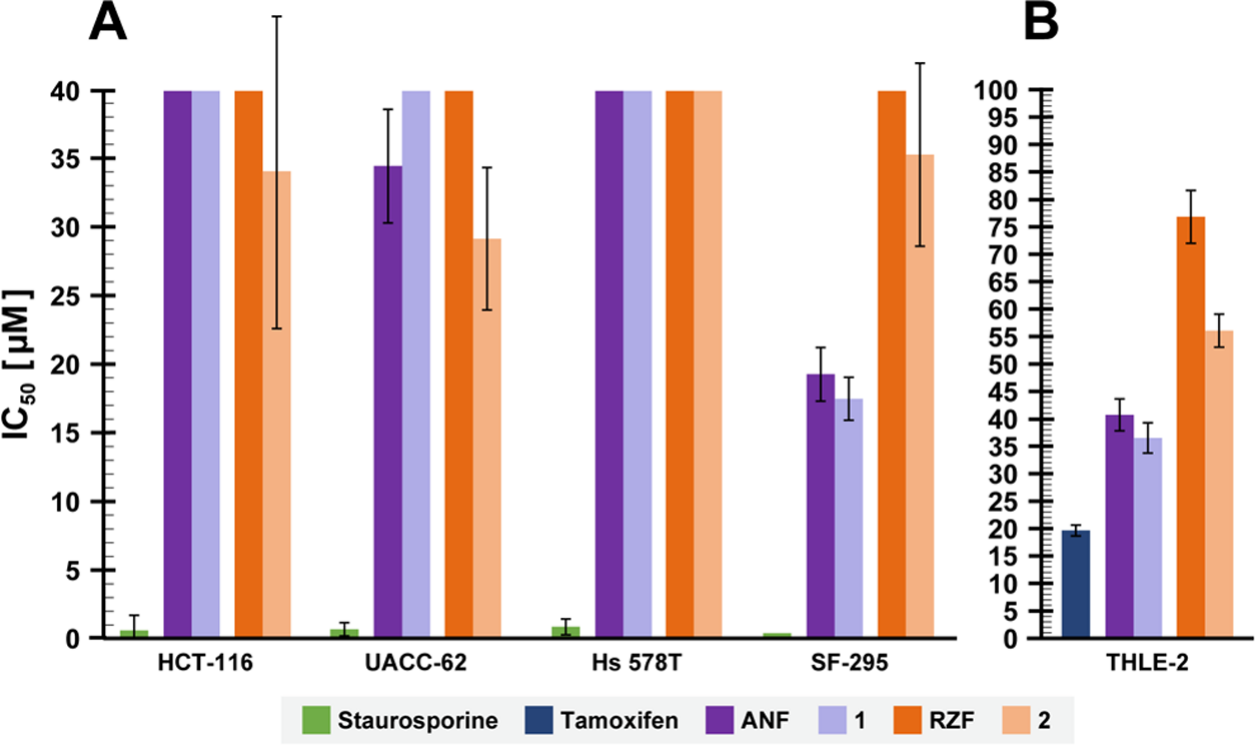
Dehydroxylation does not significantly alter the toxicity of echinocandins in human cells.^a^ (A) Effects of ANF, RZF, and dehydroxylated derivatives **1** and **2** on the viability of human-derived cell lines expressing a GFP reporter. Cells were treated with different concentrations of each compound for 24 h, and viability was determined by analyzing the fluorescent images of the cells. Each concentration was tested in triplicate, and the IC_50_ values are expressed as means ±SD. The lack of an SD bar indicates IC_50_ > 40 μM. (B) Effects of the echinocandins on the viability of THLE-2 cells. Cells were treated with different concentrations of each compound for 72 h, and viability was determined by a luminescence-based assay to determine the number of viable cells based on quantitation of the ATP present. ^a^ Each concentration was tested in triplicate.

The structures of dehydroxy-ANF (**1**) and dehydroxy-RZF (**2**) were independently determined by analyses of their one-dimensional (1D) (^1^H and ^13^C) and two-dimensional (2D) (COSY, HSQC, and HMBC) NMR spectra in CD_3_OD and by high-resolution-electrospray ionization-mass spectrometry (HR-ESI-MS). The HR-ESI-MS spectrum of compound **1** showed a molecular ion at *m*/*z* 1124.5194 corresponding to the molecular formula C_58_H_74_N_7_O_16_, suggesting that it is a dehydroxy product of ANF. Comparison of the ^1^H NMR spectrum of **1** with that of ANF (Table S1) revealed changes in the range where oxymethines appear in the spectrum and the appearance of new benzylic methylene (δ_H_ 2.65, 2.58). A comparison of the ^13^C NMR spectra revealed that one of the oxygenated methine carbons of ANF is absent in the spectrum of **1** and that a new methylene signal is present at δ_C_ 40.9. The analysis of the 2D (COSY, HSQC, and HMBC) NMR spectra of **1** (Table S1) established that the hydroxyl at position 4 of 3*S*,4*S*-dihydroxy-l-homotyrosine in ANF was substituted by a hydrogen atom to yield 3*S*-hydroxy-l-homotyrosine in **1**. The rest of the amino acids of **1** were unchanged relative to ANF. The HR-ESI-MS spectrum of compound **2** showed a molecular ion at *m*/*z* 1209.6095, corresponding to the molecular formula C_63_H_85_N_8_O_16_. The analysis of the 1D and 2D NMR spectra of **2** (Table S2) confirmed that the aminal moiety of dihydroxy ornithine of **1** had been converted into choline hemiaminal ether. The finding that compound **2** is a choline hemiaminal ether derivative of dehydroxy-ANF was further supported by comparison of ^1^H and ^13^C NMR data with those of **1** and RZF (Table S3). Comparison of the ROESY NMR spectra of compound **2** and RZF (Figures S13–S16, respectively) revealed high similarities between the sets of nuclear Overhauser effect (NOE) interactions of these two echinocandins. This supports that the two compounds have similar three-dimensional (3D) structures and that RZF has the *R*-configuration at the choline hemiaminal ether (C5 of the dihydroxy ornithine).

### Benzylic Dehydroxylation Improves the Antifungal Activity of ANF and RZF against Echinocandin-Resistant *Candida* Strains without Significantly Affecting the Viability of Human Cells

The antifungal activities of compounds **1** and **2** were evaluated against a panel of 50 strains of *C. albicans* and *C. glabrata* (Table S4), including echinocandin-resistant strains that were constructed by introducing mutations within and near the defined hotspots in the *FKS*1 and/or *FKS*2 genes.^27^ To evaluate antifungal activity, we determined MIC values. The MIC was defined as the lowest drug concentration that led to turbidity (measured at 600 nm) of less than or equal to 35% of that of the untreated culture. As controls, we tested CSF, MCF, ANF, and RZF. The results are summarized in Figure 2 and Table S5.

Compared to ANF, its derivative **1** was 16-fold more potent against 8% of the echinocandin-resistant strains, 8-fold more potent against 8% of the resistant strains, 4-fold more potent against 26% of the resistant strains, and 2-fold better against 21% of the resistant strains (Table S5). For the remaining 37% of resistant strains, the activity of derivative **1** was similar to that of the parent ANF. Removal of the benzylic alcohol from RZF had more pronounced effects: the activity of derivative **2** was 16-fold more potent against 21% of the resistant strains, 8-fold more potent in 21% of the resistant strains, 2- to 4-fold more potent against 29% of the resistant strains, and similar to that of RZF against 29% of the resistant strains (Table S5). Of note, comparison between the MIC values of the dehydroxylated derivatives versus the parent compounds revealed that the antifungal activity of **1** was improved relative to ANF against 68% of the strains and that the antifungal activity of **2** was improved relative to RZF against 72% of the strains in the entire tested panel. The results of the antifungal activity evaluation indicated that removal of the benzylic alcohol from the nonproteinogenic 3*S*,4*S*-dihydroxy-l-homotyrosine of ANF and RZF improved the efficacy against the majority of the echinocandin-resistant *Candida* pathogens in our panel. These results show that this chemical modification reduced the effects of resistance to echinocandins, resulting from a broad variety of *FKS* mutations.

We next asked if benzylic dehydroxylation of the echinocandins affects their toxicity at the cellular level. The dose-dependent effects of ANF and RZF and their dehydroxylated derivatives **1** and **2** on the viability of four human-derived cell lines expressing a green fluorescent protein (GFP) reporter protein were evaluated (Figure 3A and Table S6). In this analysis, we used HCT-116, a human colorectal carcinoma cell line; UACC-62, a human melanoma cell line; Hs 578T, a human mammary gland carcinoma cell line, and SF-295, a human glioblastoma cell line. Staurosporine, a potent and nonselective inhibitor of protein kinases, served as a positive control. The highest concentration of the echinocandins used for the cell viability tests was determined based on the reported maximal plasma concentrations (*C*_max_) of ANF and RZF measured in humans. A *C*_max_ of approximately 7 μM was measured during a pharmacokinetic study of ANF in patients with candidemia or invasive candidiasis who were treated with a 200 mg loading dose on day 1, followed by a daily 100 mg maintenance dose.^64^ In healthy volunteers treated with a single 400 mg dose of RZF, a *C*_max_ of approximately 17 μM was reached a few hours after administration.^65^ The IC_50_ values of staurosporine after 24 h of incubation ranged from 0.34 to 0.80 μM (Figure 3A and Table S6). The IC_50_ values of ANF and **1** ranged from 17 to >40 μM, and those of RZF and **2** ranged from 29 to >40 μM (Figure 3A and Table S6). Compared to RZF, a modest reduction in viability was observed in three of the four cell lines treated with **2**. No significant differences were observed between ANF and **1**. The IC_50_ values of ANF and **1** were lower than those of RZF and **2** against SF-295.

The effects of ANF and RZF and their dehydroxylated derivatives **1** and **2** on the viability of human cells were further evaluated in an assay with THLE-2 cells, which are a transformed human liver epithelial cell line. These cells express phenotypic characteristics of normal adult liver epithelial cells. Tamoxifen, an estrogen receptor modulating antitumor drug, which has been associated with hepatotoxic side effects, served as a positive control, and the results are summarized in Figure 3B. The IC_50_ value of tamoxifen after 72 h of incubation was approximately 20 μM. The IC_50_ values of ANF and **1** ranged from 36 to 41 μM and those of RZF and **2** ranged from 36 to 77 μM (Figure 3B and Table S6). The results of the cell viability assays indicate that removal of the benzylic alcohol from 3*S*,4*S*-dihydroxy-l-homotyrosine of ANF and RZF did not markedly influence the toxicities toward immortalized human cells in culture and that the effects of echinocandins on viability vary depending on the specific cell line.

### Effects of Benzylic Dehydroxylation Suggest which Amino Acids in the Fks Binding Pocket are Likely to Reside in Proximity to the l-Homotyrosine of the Drug

Based on *in silico* analyses of evolutionarily diverse fungi, Fks has 15–18 transmembrane helices, and Johnson and co-workers experimentally confirmed the existence of 13.^18^ The HS regions of Fks were predicted to reside in close proximity to the outer leaflet of the yeast cell membrane.^18^ The dominant effect of individual resistance-conferring mutations that reside in the HS regions of Fks can be explained by a model in which these regions fold together to form a single echinocandin binding site.^18^ Johnson and co-workers, therefore, suggested that, in *Saccharomyces cerevisiae*, the lipid segment of echinocandins interacts with amino acid residues located in HS3 (amino acids 690–700) of Fks1, whereas the cyclic hexapeptide backbone interacts with amino acids in HS1 and HS2 (amino acids 635–649 and 1354–1361, respectively).^18^

Access to dehydroxy derivatives **1** and **2**, which differ from the parent echinocandins ANF and RZF, respectively, solely by the removal of the benzylic alcohol of 3*S*,4*S*-dihydroxy-l-homotyrosine, allowed us to further investigate interactions between these echinocandins and Fks. We reasoned that markedly lower MIC values for the dehydroxylated compounds in echinocandin-resistant strains with a single amino acid mutation may indicate that the unmutated amino acid is in proximity to or even directly interacts with the l-homotyrosine of these drugs. In contrast, in resistant strains with a single amino acid mutation in which the MIC values were unaffected by benzylic dehydroxylation, the mutated amino acid likely does not reside in proximity to the l-homotyrosine of the drug.

**Figure 4:**
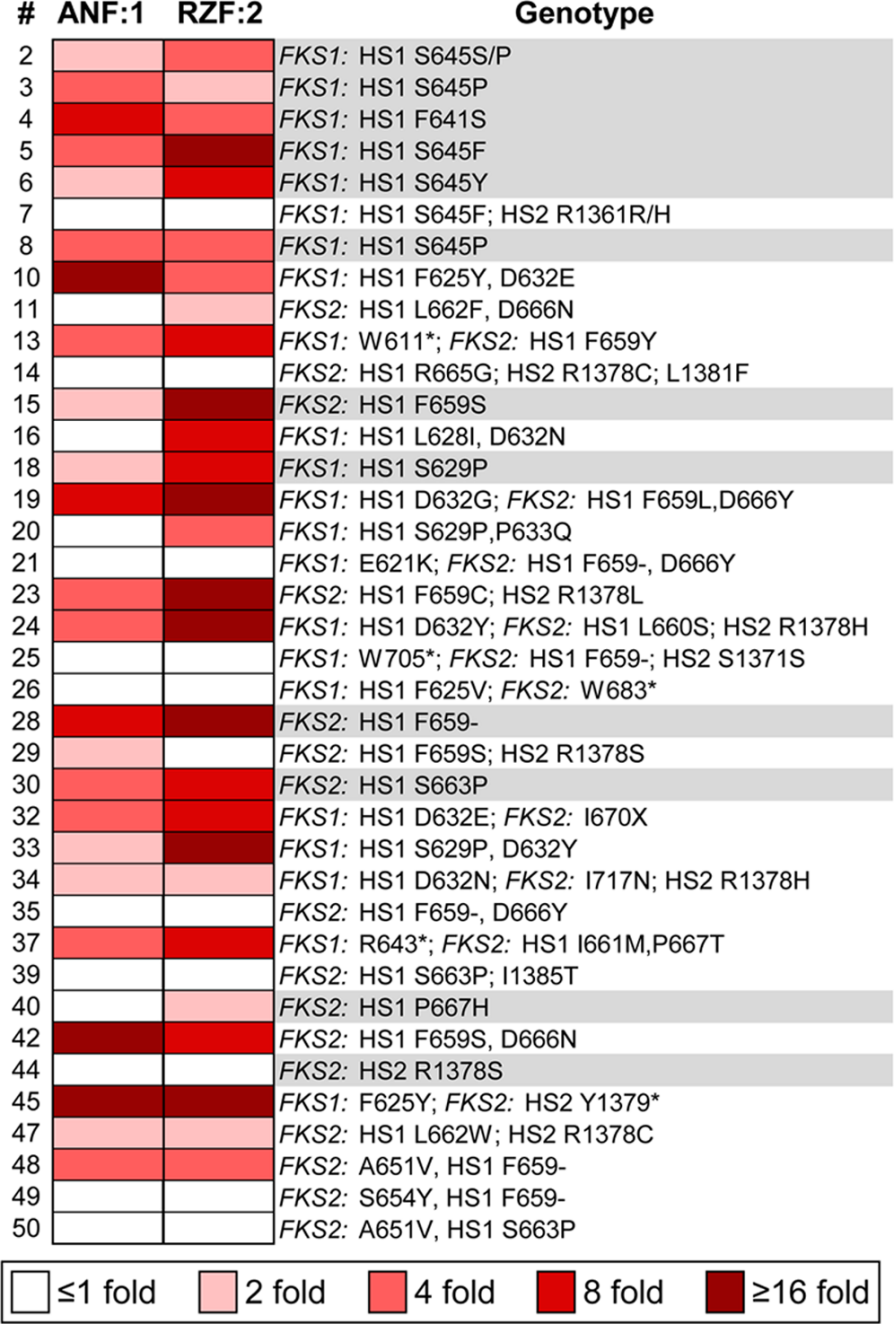
Relationships between the ratios of antifungal activities of parent echinocandins and their dehydroxylated derivatives on the *FKS* mutated strains. A dash following the amino acid number indicates a deletion, an asterisk indicates a premature termination codon, and X indicates a mutation that results in a frameshift. Ratios were determined by dividing the MIC values of the parent echinocandins by the corresponding values of the dehydroxy derivatives (ratio = MIC (ANF)/MIC (**1**) or ratio = MIC (RZF)/MIC (**2**)). Gray-colored rows indicate strains in which only a single amino acid was mutated. Strains 2–8 are echinocandin-resistant *C. albicans* and strains 10–50 are echinocandin-resistant *C. glabrata*.

In echinocandin-resistant *C. albicans* strains in the panel with single amino acid mutations (strains 2–6 and 8, Figure 4), a few amino acids stood out. The largest MIC difference between ANF and its dehydroxy derivative **1** (8-fold) was measured for *C. albicans* strain 4 in which amino acid 641, located in HS1, is serine; this residue is phenylalanine in the corresponding sensitive strain. This suggests that in *C. albicans*, phenylalanine 641 of the target Fks1 protein is in proximity to the l-homotyrosine of the bound echinocandin. The largest MIC differences (at least 8-fold) between RZF and its dehydroxy derivative **2** were measured for *C. albicans* strains 5 and 6. In these strains, serine 645 located in HS1 of Fks1 protein is replaced by phenylalanine (strain 5) or tyrosine (strain 6). This suggests that in *C. albicans*, serine 645 of Fks1 is in proximity to the l-homotyrosine of this echinocandin.

Of the *C. glabrata* strains tested, six resistant strains had single amino acid mutations (strains 15, 18, 28, 30, 40, and 44, Figure 4). The same MIC values were measured for ANF and RZF and for their dehydroxy derivatives **1** and **2**, respectively, against echinocandin-resistant *C. glabrata* strain 44 in which arginine 1378 is replaced by serine. This implies that in *C. glabrata*, arginine 1378 is not likely to reside in proximity to the l-homotyrosine of the echinocandins in this study. In *C. glabrata* strain 28, dehydroxylation of ANF resulted in an 8-fold increase in potency (Table S5). In this strain, phenylalanine 659 of the Fks2 protein is deleted, which suggests that this amino acid resides near l-homotyrosine of these echinocandins. Improvements of 8- to 16-fold in the MIC values of dehydroxy derivative **2** relative to the parent RZF were observed against four of the *C. glabrata* strains in which resistance stemmed from single mutations (strains 15, 18, 28, and 30, Figure 4), suggesting that phenylalanine 659 and serine 663 of Fks2 protein and serine 629 of Fsk1 reside in proximity to l-homotyrosine of RZF.

In an attempt to explain why the effect of benzylic dehydroxylation on echinocandin resistance was more pronounced for RZF than for ANF, we asked how this modification affects the 3D structure of these echinocandins. In 1992, Zambias and co-workers reported the solid-phase synthesis of a collection of echinocandin derivatives with a simplified hexapeptide ring.^60^ Structure–activity relationship analysis of these derivatives revealed that changes in the amino acids of the hexapeptide ring disrupted internal hydrogen bonds and reduced or abolished antifungal activity. Thus, the 3D structure of echinocandins is critical. We speculated that the removal of the benzylic alcohol of 3*S*,4*S*-dihydroxy-l-homotyrosine had a more pronounced effect on the 3D structure of ANF than RZF. To test this, we performed *in silico* density functional theory calculations on these echinocandins and their corresponding dehydroxylated derivatives **1** and **2**. Structures were optimized using the BP86 method^66,67^ with Ahlrichs’ def2-SVP basis set^68^ and the conductor-like polarizable continuum model^69,70^ to account for the water solvation effect. Atom Cartesian coordinates for RZF and ANF and their corresponding dehydroxylated derivatives **1** and **2** are provided in the Supporting Information.

**Figure 5:**
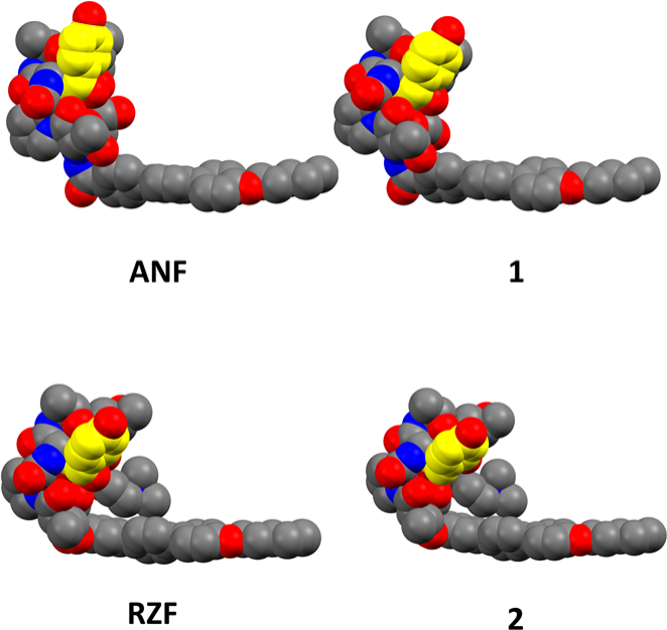
Space-filling representation of the structures of ANF, RZF, **1**, and **2** obtained using density functional theory simulations. The hydrogen atoms were omitted for clarity. Red spheres indicate oxygen atoms, blue spheres indicate nitrogen atoms, gray spheres indicate carbon atoms, and yellow spheres indicate carbon atoms of 3*S*,4*S*-dihydroxy-l-homotyrosine in ANF and RZF and the corresponding 3*S*-hydroxy-l-homotyrosine in **1** and **2**.

The computational results revealed considerable differences in the 3D structures of ANF and its dehydroxy derivative **1** (Figure 5). As speculated, the structural effects of this modification on the 3D structure of RZF were less pronounced (Figure 5). Due to the indicated changes in its conformation, ANF derivative **1** may not share the same set of interactions of ANF with the binding site in the target Fks. This may offer an explanation for the observed reduced effects of benzylic dehydroxylation of ANF on its efficacy against the echinocanin-resistant strains in the tested panel.

## Conclusions

Echinocandins are among the latest additions to the limited arsenal of antifungal agents in clinical use. These agents are the recommended first-line medications for patients with invasive fungal infections such as candidiasis. The increase in echinocandin resistance among pathogenic fungi, especially among strains of the genus *Candida*, the most common human fungal pathogens, is a great concern. The only echinocandin resistance mechanism known to date stems from mutations in “hot spot” regions in *FKS* genes, which encode the catalytic subunit of the GS complex.

Our study reports the first example of a chemical modification that restores the efficacy of echinocandins against strains that have evolved mutations in *FKS* genes that reduce the affinity of echinocandins for their target Fks protein. One-step chemoselective benzylic dehydroxylation to convert 3*S*,4*S*-dihydroxy-l-homotyrosine, one of the nonproteinogenic amino acids in the cyclic hexapeptide segment of echinocandins, to the corresponding 3*S*-hydroxy-l-homotyrosine was carried out on the drug ANF to afford the corresponding novel dehydroxy echinocandin **1**. Compound **2**, the dehydroxy derivative of RZF, a novel once-weekly administered echinocandin currently being tested in phase III clinical trials, was generated from **1** in a single step by conversion of its hemiaminal to the corresponding choline hemiaminal ether.

The 3*S*-hydroxy-l-homotyrosine derivatives of ANF and RZF, compounds **1** and **2**, respectively, had improved efficacy against the majority of the echinocandin-resistant *Candida* pathogens in the tested panel. To date, no ternary structure of the target Fks protein bound to an echinocandin has been reported. As the tested panel included a collection of echinocandin-resistant *Candida* strains constructed by introducing single mutations in the *FKS*1 and/or *FKS*2 genes, analyses of strain susceptibilities to the dehydroxylated derivatives suggested that certain amino acids of Fks proteins likely reside in proximity to l-homotyrosine of the cyclic hexapeptide segment of the echinocandins in this study. The largest improvements in antifungal activities of the dehydroxy derivatives relative to those of the parent echinocandins were observed in *C. albicans* strains with mutations at phenylalanine 641 and serine 645 located in HS1 of Fks1. In *C. glabrata*, the largest improvements were observed in strains with mutations at serine 629 of HS1 of Fks1 protein and phenylalanine 659 and serine 663 located in HS1 of the Fks2 protein. These results suggest that 3*S*,4*S*-dihydroxy-l-homotyrosine of ANF and RZF may reside in proximity, or directly interact, with these amino acids.

To conclude, this study establishes a robust chemical modification avenue that restores the efficacy of echinocandin drugs against a large percentage of strains with known resistance-causing mutations. This work paves the way for the development of novel members of this important antifungal drug class.

## Materials and Methods

### General Chemistry Methods and Instrumentation

The 1D ^1^H- and ^13^C NMR spectra and the 2D-COSY, HSQC, HMBC, and ROESY experiments were recorded on a Bruker Avance III 400 or 500 spectrometer operating at 400 or 500 MHz, respectively, for ^1^H and at 100 or 125 MHz, respectively, for ^13^C. Chemical shifts (reported in ppm) were calibrated to CD_3_OD (^1^H: δ = 3.31,^13^C: δ = 49.0). High-resolution electrospray ionization-mass spectra (HR-ESI-MS) were measured on a Waters Synapt instrument. Low-resolution ESI-MS were measured on a Waters SQD-2 mass detector. All chemicals, unless otherwise stated, were obtained from commercial sources. The preparative reversed-phase high-performance liquid chromatography (RP-HPLC) system used was an ECOM system equipped with a 5 μm, C-18 Phenomenex Luna Axia column (250 mm × 21.2 mm). Analytical RP-HPLC was performed on a VWR Hitachi instrument equipped with a diode array detector and an Alltech Apollo C18 reversed-phase column (5 μm, 4.6 mm × 250 mm). The flow rate was 1 mL/min. Solvent A was 0.1% TFA in water and solvent B was acetonitrile.

### Synthesis of Compound **1**

ANF (103 mg, 0.09 mmol) was dissolved in THF (10 mL) and treated with palladium hydroxide on carbon (20 wt %, matrix carbon, wet support, 300 mg) and hydrogen (balloon). The reaction was allowed to stir at ambient temperature, and progress was monitored by analytical RP-HPLC (acetonitrile in H_2_O containing 0.1% TFA; gradient from 10 to 90%; flow rate: 1 mL/min). Approximately, 50% formation of dehydroxylated compound **1** was observed after 2 days. The reaction mixture was then filtered (0.22 μm), and the solvent was removed by evaporation. The crude product was dissolved in methanol and purified by preparative RP-HPLC (mobile phase: acetonitrile in H_2_O containing 0.1% TFA; gradient from 50 to 95%; flow rate: 15 mL/min) to yield 34 mg of compound **1** as a white powder (33% isolated yield, ≥95% pure as determined by RP-HPLC, Figure S13). HR-ESI-MS *m*/*z* calculated for C_58_H_74_N_7_O_16_, 1124.5192; found [M + H]^+^, 1124.5194.

### Synthesis of Compound **2**

Compound **1** (73 mg, 0.06 mmol, 1 equiv) was dissolved in dry DMSO (2 mL) under an argon atmosphere, and choline chloride (720 mg, 80 equiv) and 4 M HCl in dioxane (40 μL, 2.7 equiv) were added. The reaction was allowed to stir at ambient temperature, and progress was monitored by analytical RP-HPLC (acetonitrile in H_2_O containing 0.1% TFA; gradient from 10 to 90%; flow rate: 1 mL/min). Approximately, 38% conversion was observed after 2 days. The reaction mixture was then diluted with acetonitrile/H_2_O (1:1) and purified by preparative RP-HPLC (mobile phase: acetonitrile in H_2_O containing 0.1% TFA; gradient from 10 to 90%; flow rate: 15 mL/min) to yield 28 mg of compound **2** as a white powder (36% isolated yield, ≥95% pure as determined by RP-HPLC, Figure S14). HR-ESI-MS *m*/*z* calculated for C_63_H_85_N_8_O_16_, 1209.6084; found [M]^+^, 1209.6095.

### *Candida* Strains

The laboratory and clinical isolates and ATCC strains used in this study are listed in Table S4. Three *FKS* mutants derived from *C. albicans* SC5314,^39,71^ four clinical isolates,^39^ and a collection of 31 *FKS* mutants derived from 11 different genetic backgrounds of *C. glabrata* strains^27,72^ were used in this study.

### Minimum Inhibitory Concentration Broth Double-Dilution Assay

All of the tested compounds were dissolved in anhydrous DMSO to a concentration of 5 mg/mL. *Candida* strains were streaked from a glycerol stock onto YPAD agar plates and grown for 24 h at 30 °C. Colonies were suspended in 1 mL of PBS and diluted to an O.D. of 0.01 at 600 nm with YPAD broth and added into flat-bottom 96-well microplates (Corning) containing a gradient of twofold dilutions per tested compound with concentrations ranging from 64 to 0.0009 μg/mL. Control wells with no drug and blank wells without yeast cells containing YPAD only were also prepared. MIC values (Table S5) were determined after 24 h at 30 °C by measuring the optical density at 600 nm using a plate reader (Tecan Infinite M200 PRO). MIC values were defined as the point at which the optical density was reduced by ≥65% compared to the no-drug wells. Each concentration was tested in triplicate, and results were confirmed by at least two independent sets of experiments.

### Phenotypic Viability Assay of Human-Derived Cell Lines Expressing a GFP Reporter Protein

Human cell lines HCT-116, UACC-62, Hs 578T, and SF-295 were obtained from the Life Sciences Core Facilities of the Nancy & Stephen Grand Israel National Center for Personalized Medicine. Cell lines were grown in an RPMI medium (Gibco, cat. no. 21875-034) supplemented with 10% fetal calf serum (Biological Industries, cat. no. 04-007-1A), Pen/Strep (Biological Industries, cat. no. 03-031-1B), and glutamine (Biological Industries, cat. no. 03-020-1B). GFP was introduced into cells using TALENS according to a previously reported protocol.^73^ GFP cells were selected by FACS and propagated.

Assay-ready plates were designed using in-house software (CHEMPION, INCPM software development team) and prepared in 384-well Greiner plates (cat. no. 781091) using an Echo 555 Acoustic Liquid Handler (Labcyte). The final DMSO concentration in all samples was 0.4%. Prior to the experiment, cells were trypsinized and counted using a Countess cytometer. Cells were then diluted to 40,000 cells/mL in a fresh medium. Cells (50 μL/well; 2000 cells/well) were then dispensed with a Thermo Combi dispenser into plates containing predispensed compounds. After 30 min at room temperature to ensure uniform adherence of cells, the plates were moved to an automated incubator (Liconic STX44) and incubated at 37 °C. An automated confocal microscope with a 4× S Fluor lens (Molecular Devices ImageExpress) was used to acquire images of GFP-expressing cells after 24 h of incubation. Images were then analyzed using MetaExpress with a custom protocol (Custom Module Editor), and data were annotated and normalized using Genedata Screener software. Final normalized data were then loaded into CDD Vault software for comparative data visualization.

### Viability Assay of THLE-2 Cells

THLE-2 cells (ATCC, cat. no. CRL2706) were cultured in 384-well plates coated with BEBM (Lonza, cat. no CC3170) containing 100 μg/mL BSA, 1:100 fibronectin (Sigma, cat. no. F0895), and a 1:100 PureCol EZ Gel Solution (Sigma, cat. no. 5074) for 50–60 min at 37 °C. THLE-2 cells were plated at a density of 2.5 × 10^3^ cells/well in the coated plates in a total volume of 20 μL. Culture media were BEGM supplemented with 5 ng/mL hEGF (Sigma, cat. no. E5036), 70 ng/mL phosphoethanolamine (Sigma, cat. no. P0503), and 10% fetal bovine serum. Plates were incubated overnight at 37 °C, 95% humidity, and 5% CO_2_. The culture medium was replaced with 50 μL of a fresh medium, and compounds were applied to the cells in triplicate. Plates were cultured for another 72 h, and ATP was quantified using a CellTiter-Glo kit (Promega #G7573) according to the manufacturer’s protocol. The luminescence signal was detected using a PHERAstar FS (BMG Labtech).

## Supporting Information Available

Detailed ^1^H and ^13^C NMR assignments and COSY and HMBC correlations (Tables S1–S3); yeast strain information (Table S4); MIC values (Table S5); ^1^H NMR,^13^C NMR, 2D-HSQC, 2D-COSY, 2D-HMBC, and 2D-ROESY spectra (Figures S1–S16); analytic HPLC chromatograms (Figures S17 and S18); and density functional theory (DFT) calculation data (PDF)
