# Supplementary material for: Benzylic Dehydroxylation of Echinocandin Antifungal Drugs Restores Efficacy against Resistance Conferred by Mutated Glucan Synthase
Source: J Am Chem Soc. 2022 Mar 29;144(13):5965–75. doi: 10.1021/jacs.2c00269 (PMC8991007; doi:10.1021/jacs.2c00269)
Supplement: Supplementary file 1 — ja2c00269_si_001.pdf [file ja2c00269_si_001.pdf]

Supporting Information

Benzylic Dehydroxylation of Echinocandin Antifungal Drugs Restores Efficacy Against Resistance Conferred by Mutated Glucan Synthase

Dana Logviniuk,[a] Qais Z. Jaber, [a] Roman Dobrovetsky,[a] Noga Kozer,[b] Ewa Ksiezopolska,[c,d] Toni Gabaldón,[c,d,e,f] Shmuel Carmeli, [a] and Micha Fridman[a,]*

[a] School of Chemistry, Raymond & Beverly Sackler Faculty of Exact Sciences, Tel Aviv University, Tel Aviv, 6997801, Israel.

[b] The Wohl Drug Discovery institute of the Nancy and Stephen Grand Israel National Center for Personalized Medicine, Weizmann Institute of Science, Rehovot, 7610001, Israel.

[c] Barcelona Supercomputing Centre (BSC-CNS), Jordi Girona, 29, Barcelona, 08034, Spain.

[d] Institute for Research in Biomedicine (IRB Barcelona), The Barcelona Institute of Science and Technology, Baldiri Reixac, 10, Barcelona, 08028, Spain.

[e] Catalan Institution for Research and Advanced Studies (ICREA), Passeig de Lluís Companys, 23, Barcelona, 08010, Spain.

[f] Centro Investigación Biomédica En Red de Enfermedades Infecciosas, Madrid, 28029, Spain.

*E-mail: mfridman@tauex.tau.ac.il; Phone: (+972)-3-6408687

1. CHEMISTRY

1.1. NMR data

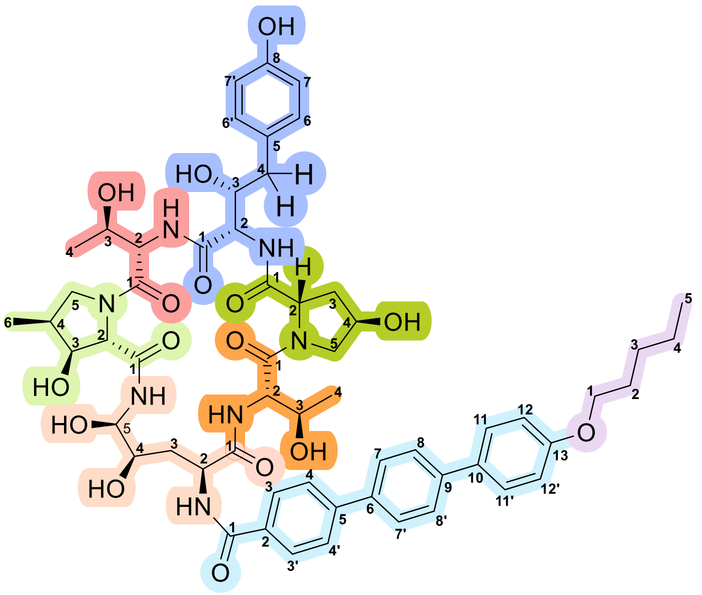

1

Table S1. NMR data of compound **1** in CD_3OD.[a]

Position	δ_C, mult.[b]	δ_H, mult.[b] (*J* in Hz)	COSY correlations	HMBC correlations[c]
diOHOrn-1	174.3, C			diOHOrn-2,3a,3b, Thr2-2
diOHOrn-2	52.5, CH	4.66, dd (4.8, 2.8)	diOHOrn-2-NH, 3a,3b	diOHOrn-3a,3b,
diOHOrn-3a	34.9, CH_2	2.23, ddd (13.9, 8.6, 4.8)	diOHOrn-2,3b,4	diOHOrn-2,4
-3b		2.14, ddd (13.9, 6.0, 2.8)	diOHOrn-2,3a,4	
diOHOrn-4	71.2, CH	4.10, ddd (8.6, 6.0, 2.5)	diOHOrn-3a,3b,5	diOHOrn-3a,3b
diOHOrn-5	74.6, CH	5.39, d (2.5)	diOHOrn-4,5-NH	diOHOrn-3a,3b
diOHOrn-2-NH		8.60, d (7.3)*	diOHOrn-2	
diOHOrn-5-NH		8.44, 1d (8.0)*	diOHOrn-5	
TPh-1	169.9, C			diOHOrn-2, TPh-3,3',4,4'
TPh-2	133.8, C			TPh-4,4'
TPh-3,3'	129.2, CHx2	7.95, d (8.3)	TPh-4,4'	TPh-3,3',4,4'
TPh-4,4'	127.6, CHx2	7.75, d (8.3)	TPh-3,3'	TPh-3,3',4,4'
TPh-5	145.3, C			TPh-3,3',4,4'
TPh-6	139.3, C			TPh-4,4',8,8'
TPh-7,7'	128.5, CHx2	7.73, d (8.3)	TPh-8,8'	TPh-7,7'
TPh-8,8'	128.0, CHx2	7.68, d (8.3)	TPh-7,7'	TPh-7,7',8,8'
TPh-9	141.8, C			TPh-7,7',11,11'
TPh-10	133.9, C			TPh-8,8',11,11'
TPh-11,11'	128.9, CHx2	7.60, d (9.0)	TPh-12,12'	TPh-11,11'
TPh-12,12'	115.9, CHx2	7.00, d (9.0)	TPh-11,11'	TPh-11,11',12,12'
TPh-13	160.6, C			TPh-11,11',12,12', *O*-pent-1
O-pent-1	69.1, CH_2	4.02, t (6.4)	*O*-pent-2	*O*-pent-2,3
O-pent-2	30.1, CH_2	1.81, tt (6.4, 7.3)	*O*-pent-1,3	*O*-pent-1,3

O-pent-3	29.4, CH_2	1.49, m	*O*-pent-2,4	*O*-pent-1,3,4,5
O-pent-4	23.5, CH_2	1.43, m	*O*-pent-3,5	*O*-pent-2,3,5
O-pent-5	14.4, CH_3	0.97, t (7.3)	*O*-pent-4	*O*-pent-3,4
OHMePro-1	172.6, C			diOHOrn-5, OHMePro-2
OHMePro-2	69.7, CH	4.38, d (2.9)	OHMePro-3	OHMePro-3,5a
OHMePro-3	75.7, CH	4.21, dd (2.9, 4.7)	OHMePro-2,4	OHMePro-2,5a,5b,6
OHMePro-4	39.1, CH	2.55, m	OHMePro-3,5a,5b,6	OHMePro-2,3,5a,5b,6
OHMePro-5a	52.9, CH_2	3.89, dd (9.3, 7.7)	OHMePro-4,5b	OHMePro-3,4,6
-5b		3.42, t (9.3)	OHMePro-4,5a	
OHMePro-6	11.3, CH_3	1.08, d (6.9)	OHMePro-4	OHMePro-4,5a,5b
Thr1-1	170.0, C			Thr1-2,3
Thr1-2	56.7, CH	4.90, m	Thr1-2-NH,3	Thr1-4
Thr1-3	69.5, CH	4.23, dq (4.4, 6.6)	Thr1-2,4	Thr1-2,4
Thr1-4	20.3, CH_3	1.23, d (6.6)	Thr1-3	Thr1-2,3
Thr1-NH		7.60, m	Thr1-2	
OHHty-1	172.7, C			Thr1-2, OHHty-2,3
OHHty-2	57.4, CH	4.53, brd (1.1)	OHHty-2-NH,3	OHHty-4a,4b
OHHty-3	74.6, CH	4.37, m	OHHty-2,4a,4b	OHHty-2,4a,4b
OHHty-4a	40.9, CH	2.65, dd (13.6, 6.5)	OHHty-3,4b	OHHty-3,5,5’
-4b		2.58, dd (13.6, 7.5)	OHHty-3,4a	
OHHty-5	129.8, C			OHHty-3,4a,4b,7,7’
OHHty-6,6’	131.6, CHx2	7.03, d (8.7)	OHHty-7,7’	OHHty-4a,4b,6,6’
OHHty-7,7’	116.3, CHx2	6.71, d (8.7)	OHHty-6,6’	OHHty-6,6’,7,7’
OHHty-8	157.1, C			OHHty-6,6’,7,7’
OHHty-2-NH		7.69, m*	OHHty-2	
OHHty-8-OH		9.07 s*		
OHPro-1	174.1, C			OHHty-2, OHPro-2
OHPro-2	62.5, CH	4.67, dd (7.1, 3.3)	OHPro-3a,3b	OHPro-4,3b,5b
OHPro-3a	38.9, CH_2	2.48, dd (13.0, 7.1)	OHPro-2,3b,4	OHPro-2,5b
-3b		2.08, dt (3.3, 13.0)	OHPro-2,3a,4	
OHPro-4	71.3, CH	4.58, brm	OHPro-3a,3b,5a,5b	OHPro-2,3a,5b
OHPro-5a	57.2, CH_2	4.04, dd (11.0, 3.1)	OHPro-4,5b	OHPro-4,3a
-5b		3.83, brd (11.0)	OHPro-4,5a	
Thr2-1	172.9, C			OHPro-2,5b, Thr2-2
Thr2-2	58.9, CH	5.03, d (2.8)	Thr2-2-NH,4	Thr2-4
Thr2-3	68.4, CH	4.56, dq (2.8, 6.2)	Thr2-2,4	Thr2-2,4
Thr2-4	19.5, CH_3	1.27, d (6.2)	Thr2-3	Thr2-3
Thr2-2-NH		8.66, d (9.0)*	Thr2-2	

[a]500 MHz for ^{1}H, 125 MHz for ^{13}C. [b]Multiplicity, [c]Correlations of protons quoted to carbon in line. *Residual signal. Abbreviations of amino acids and other regions in the molecule: diOHOrn, 4,5-L-dihydroxyornithine; TPh, triphenyl; *O*-pent, *O*-pentyl; OHMePro, 3-hydroxy-4-methyl-L-proline; Thr, L-threonine; OHHty, 3-hydroxy-L-homotyrosine; OHPro, 4-hydroxy-L-proline.

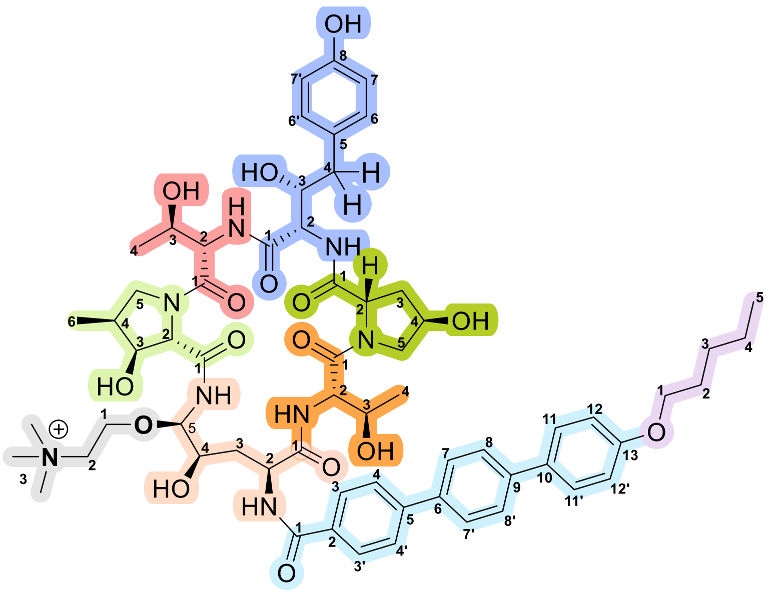

2

Table S2. NMR data of compound **2** in CD_3OD.[a]

Position	δ_C, mult[b]	δ_H, mult[b] (J in Hz)	COSY correlations	HMBC correlations[c]
OHOrn-1	173.7, C			OHOrn-2,3b
OHOrn-2	51.7, CH	4.76, dd (12.4,5.1)	OHOrn-3a,3b	OHOrn-3b
OHOrn-3	36.2, CH_2	2.30, ddd (14.0,9.7,5.1)	OHOrn-2,3b,4	OHOrn-2
		2.07, m	OHOrn-2,3a,4	
OHOrn-4	70.0, CH	4.10, ddd (8.6,5.9,1.3)	OHOrn-3a,3b,5	OHOrn-2,3a,3b
OHOrn-5	81.2, CH	5.48, brd (1.3)	OHOrn-4,5-NH	OHOrn-3a,3b,4, Chol-1a,1b
OHOrn-2-NH		8.49, d (8.0)*	OHOrn-2	
OHOrn-5-NH		8.46, d (9.4)*	OHOrn-5	
Tph-1	169.5, C			Tph-3,3',4,4' OHOrn-2
Tph-2	133.7, C			Tph-4,4'
Tph-3,3'	129.2, CH x2	7.97, d x2 (8.4)	Tph-4,4'	Tph-3,3',4,4'
Tph-4,4'	127.9, CH x2	7.79, d x 2 (8.4)	Tph-3,3'	Tph-3,3',4,4'
Tph-5	145.5, C			Tph-3,3',4,4'
Tph-6	139.1, C			Tph-4,4',8,8'
Tph-7,7'	128.5, CH x2	7.75, d x 2 (8.8)	Tph-8,8'	Tph-7,7',8,8'
Tph-8,8'	128.0, CH x2	7.70, d x 2 (8.8)	Tph-7,7'	Tph-7,7'
Tph-9	142.0, C			Tph-7,7',11,11'
Tph-10	133.9, C			Tph-8,8'12,12'
Tph-11,11'	128.9, CH x2	7.60, d x 2 (8.8)	Tph-12,12'	Tph-11,11'
Tph-12,12'	116.0, CH x2	7.00, d x 2 (8.8)	Tph-11,11'	Tph-12,12'
Tph-13	160.5, C			Tph-11,11',12,12', *O*-pent-1
O-pent-1	69.1, CH_2	4.03, t (6.4)	*O*-pent-2	*O*-pent-2,3
O-pent-2	30.2, CH_2	1.81, tt (7.3,6.4)	*O*-pent-1,3	*O*-pent-1,3,4
O-pent-3	29.4, CH_2	1.49, m	*O*-pent-2,4	*O*-pent-1,2,4
O-pent-4	23.6, CH_2	1.42, m	*O*-pent-3,5	*O*-pent-2,3,5
O-pent-5	14.4, CH_3	0.97, t (7.2)	*O*-pent-4	*O*-pent-3,4

Chol-1a	62.7, CH_2	4.03, m	Chol-1b,2a,2b	OHOrn-5, Chol-2a,2b
-1b		3.93, m	Chol-1a,2a,2b	
Chol-2a	66.7, CH_2	3.62, ddd (14.1,7.5,2.4)	Chol-2b	Chol-3-Me_3
-2b		3.54, ddd (14.1,5.9,2.4)	Chol-2a	
Chol-3-Me_3	54.7, CH_3	3.14, s x 3		Chol-1a,1b,3-Me_3
OHMePro-1	174.3, C			OHOrn-5, OHMePro-2,3
OHMePro-2	68.3, CH	4.40, d (4.2)	OHMePro-3	OHMePro-5a,5b
OHMePro-3	76.3, CH	4.25, t (4.2)	OHMePro-2,4	OHMePro-2,5a,5b,6
OHMePro-4	39.0, CH	2.51, m	OHMePro-3,5a,5b,6	OHMePro-2,5a,5b,6
OHMePro-5a	53.3, CH_2	3.90, dd (10.0,7.1)	OHMePro-4,5b	OHMePro-3,4,6
b		3.50, dd (10.0,7.0)	OHMePro-4,5a	
OHMePro-6	11.6, CH_3	1.09, d (6.8)	OHMePro-5	OHMePro-3,4,5a,5b
Thr1-1	170.1, C			OHMePro-2, Thr1-2,3
Thr1-2	56.8, CH	4.90, d (4.7)	Thr1-3	Thr1-4
Thr1-3	69.5, CH	4.18, dd (6.4,4.7)	Thr1-2,4	Thr1-2,4
Thr1-4	20.2, CH_3	1.24, d (6.0)	Thr1-3	Thr1-2,3
Thr1-2-NH		7.59, m*	Thr1-2	
OHHty-1	172.8, C			Thr1-2, OHHty-2
OHHty-2	57.2, CH	4.53, brd (1.4)	OHHty-3	OHHty-4a,4b
OHHty-3	74.6, CH	4.37, m	OHHty-2,4a,4b	OHHty-4a,4b
OHHty-4a	40.9, CH_2	2.65, dd (13.6,6.6)	OHHty-3,4b OHHty-3,4a	OHHty-6,6’
4b		2.59, dd (13.6,7.6)		
OHHty-5	129.6, C			OHHty-3,4a,4b,7,7’
OHHty-6,6’	131.5, CH x2	7.02, d x 2 (8.8)	OHHty-7,7’	OHHty-4a,4b,6,6’
OHHty-7,7’	116.3, CH x2	6.71, d x 2 (8.8)	OHHty-6,6’	OHHty-7’,7
OHHty-8	157.1, C			OHHty-6,6’,7,7’
OHHty-2-NH		7.63, m*	OHHty-2	
OHHty-8-OH		9.07, s*		
OHPro-1	174.0, C			OHHty-2, OHPro-2
OHPro-2	62.6, CH	4.64, dd (11.3,7.1)	OHPro-3a,3b	OHPro-3b,4,5b
OHPro-3a	39.0, CH_2	2.48, m	OHPro-2,3b	OHPro-2,5b
3b		2.08, m	OHPro-2,3a	
OHPro-4	71.3, CH	4.59, m	OHPro-5a,5b	OHPro-3a,5b
OHPro-5a	57.2, CH_2	4.03, dd (11.0,3.4)	OHPro-4,5b OHPro-4,5a	OHPro-3a
5b		3.83, d (11.0)		
Thr2-1	172.9, C			Thr2-2
Thr2-2	58.9, CH	5.06, d (2.7)	Thr2-2,3	Thr2-4
Thr2-3	68.1, CH	4.58, brs	Thr2-2,4	Thr2-2,4
Thr2-4	19.5, CH_3	1.24, d (6.0)	Thr2-3	Thr2-2
Thr2-2-NH		8.54, d (8.5)*	Thr2-2	

[a]400 MHz for ^{1}H, 100 MHz for ^{13}C. [b]Multiplicity, [c]Correlations of protons quoted to carbon in line. *Residual signal. Abbreviations of amino acids and other regions in the molecule: OHOrn, 4-hydroxy-L-ornithine; TPh, triphenyl; *O*-pent, *O*-pentyl; OHMePro, 3-hydroxy-4-methyl-L-proline; Thr, L-threonine; OHHty, 3-hydroxy-L-homotyrosine; OHPro, 4-hydroxy-L-proline, Chol, Choline.

Table S3. Comparison between the ^1H and ^{13}C NMR data of ANF, compound **1**, compound **2** and RZF in CD_3OD.

Position	ANF		compound **1**[a]		compound **2**[a]		RZF[b]	
	δ_C	δ_H	δ_C	δ_H	δ_C	δ_H	δ_C	δ_H
diOHOrn/OHOrn-1	174.4		174.3		173.7		173.8	
diOHOrn/OHOrn-2	52.7	4.67	52.5	4.66	51.7	4.76	51.9	4.76
diOHOrn/OHOrn-3a	34.9	2.23	34.9	2.23	36.2	2.30	36.1	2.29
-3b		2.12		2.14		2.07		2.05
diOHOrn/OHOrn-4	71.3	4.07	71.2	4.10	70.0	4.10	70.0	4.10
diOHOrn/OHOrn-5	74.8	5.35	74.6	5.39	81.2	5.48	81.4	5.44
diOHOrn/OHOrn-2-NH		8.58		8.60		8.49		8.49
diOHOrn/OHOrn-5-NH		8.41		8.44		8.46		8.43
TPh-1	169.9, C		169.9		169.5		169.5	
TPh-2	133.8		133.8		133.7		133.6	
TPh-3,3’	129.2	7.94	129.2	7.95	129.2	7.97	129.1	7.96
TPh-4,4’	127.7	7.75	127.6	7.75	127.9	7.79	127.8	7.78
TPh-5	145.3		145.3		145.5		145.5	
TPh-6	139.3		139.3		139.1		139.1	
TPh-7,7’	128.5	7.73	128.5	7.73	128.5	7.75	128.5	7.74
TPh-8,8’	128.0	7.69	128.0	7.68	128.0	7.70	128.0	7.70
TPh-9	141.9		141.8		142.0		142.0	
TPh-10	133.9		133.9		133.9		133.8	
TPh-11,11’	128.9	7.60	128.9	7.60	128.9	7.60	128.9	7.60
TPh-12,12’	115.9	7.00	115.9	7.00	116.0	7.00	116.0	7.00
TPh-13	160.4		160.6		160.5		160.5	
O-pent-1	69.1	4.02	69.1	4.02	69.1	4.03	69.1	4.01
O-pent-2	30.2	1.81	30.1	1.81	30.2	1.81	30.1	1.80
O-pent-3	29.4	1.49	29.4	1.49	29.4	1.49	29.4	1.49
O-pent-4	23.6	1.43	23.5	1.43	23.6	1.42	23.5	1.44
O-pent-5	14.4	0.97	14.4	0.97	14.4	0.97	14.4	0.96
Chol-1a					62.7	4.03	62.7	4.04
-1b						3.93		3.94
Chol-2a					66.7	3.62	66.7	3.62
-2b						3.54		3.52
Chol-3-Me_3					54.7	3.14	54.7	3.13
OHMePro-1	172.5		172.6		174.3		174.3	
OHMePro-2	69.6	4.37	69.7	4.38	68.3	4.40	69.8	4.34
OHMePro-3	75.6	4.22	75.7	4.21	76.3	4.25	76.2	4.26
OHMePro-4	39.1	2.54	39.1	2.55	39.0	2.51	38.9	2.51
OHMePro-5a	53.0	3.89	52.9	3.89	53.3	3.90	53.3	3.90
-5b		3.41		3.42		3.50		3.48
OHMePro-6	11.3	1.06	11.3	1.08	11.6	1.09	11.6	1.07
Thr1-1	170.0		170.0		170.1		170.1	
Thr1-2	57.2	4.87	56.7	4.90	56.8	4.90	57.0	4.88
Thr1-3	69.7	4.21	69.5	4.23	69.5	4.18	70.0	4.18
Thr1-4	20.1	1.26	20.3	1.23	20.2	1.24	20.0	1.27
Thr1-2-NH		7.58		7.60		7.59		7.58
diOHHty/OHHty-1	172.5		172.7		172.8		172.7	
diOHHty/OHHty-2	56.3	4.33	57.4	4.53	57.2	4.53	56.2	4.34

diOHHty/OHHty-3	77.0	4.25	74.6	4.37	74.6	4.37	77.1	4.25
diOHHty/OHHty-4/4a<br>-4b	75.8	4.32	40.9	2.65<br>2.58	40.9	2.65<br>2.59	75.7	4.35
diOHHty/OHHty-5	133.1		129.8		129.6		133.0	
diOHHty/OHHty-6,6’	129.6	7.15	131.6	7.03	131.5	7.02	129.6	7.15
diOHHty/OHHty-7,7’	116.2	6.77	116.3	6.71	116.3	6.71	116.2	6.77
diOHHty/OHHty-8	158.0		157.1		157.1		158.5	
diOHHty/OHHty-2-NH		7.69		7.69		7.63		7.32
OHPro-1	173.5		174.1		174.0		173.5	
OHPro-2	62.5	4.61	62.5	4.67	62.6	4.64	62.6	4.59
OHPro-3a<br>-3b	38.6	2.44<br>2.08	38.9	2.48<br>2.08	39.0	2.48<br>2.08	38.6	2.44<br>2.08
OHPro-4	71.2	4.57	71.3	4.58	71.3	4.59	71.3	4.56
OHPro-5a<br>-5b	56.9	3.98<br>3.83	57.2	4.04<br>3.83	57.2	4.03<br>3.83	57.1	3.99<br>3.81
Thr2-1	172.8		172.9		172.9		172.7	
Thr2-2	58.7	5.02	58.9	5.03	58.9	5.06	58.8	5.05
Thr2-3	68.4	4.56	68.4	4.56	68.1	4.58	68.2	4.58
Thr2-4	19.7	1.29	19.5	1.27	19.5	1.24	19.7	1.27
Thr2-2-NH		8.66		8.66		8.54		8.56

[a]500 MHz for ^{1}H, 125 MHz for ^{13}C. [b]400 MHz for ^{1}H, 100 MHz for ^{13}C. Abbreviations of amino acids and other regions in the molecule: diOHOrn, 4,5-L-dihydroxyornithine; OHOrn, 4-hydroxy-L-ornithine; TPh, triphenyl; *O*-pent, *O*-pentyl; OHMePro, 3-hydroxy-4-methyl-L-proline; Thr, L-threonine; diOHHty, 3,4-dihydroxy-L-homotyrosine, OHHty, 3-hydroxy-L-homotyrosine; OHPro, 4-hydroxy-L-proline, Chol, Choline.

1.2. NMR spectra

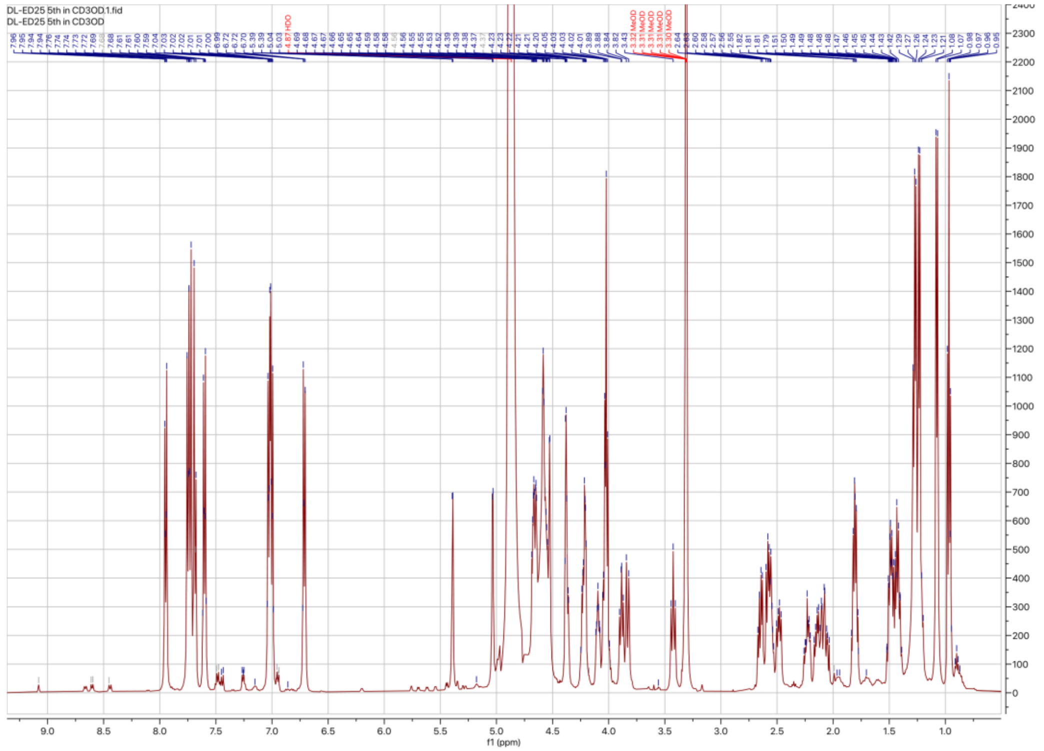

Figure S1. 500 MHz ^{1}H-NMR spectrum of compound **1** in CD_3OD.

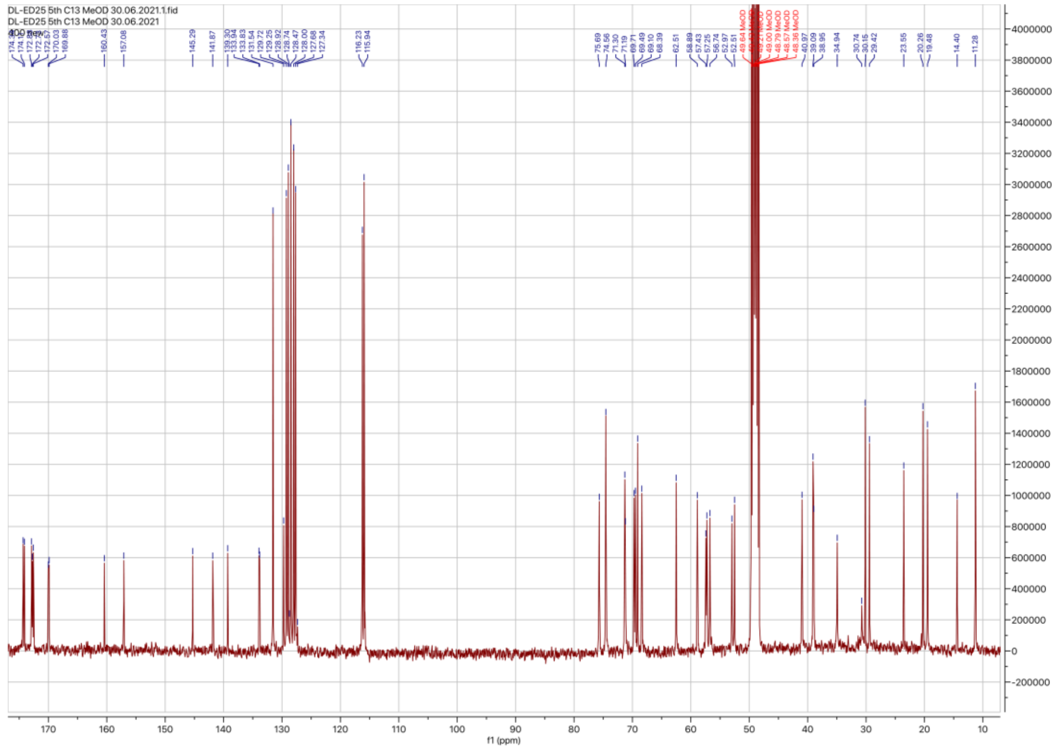

Figure S2. 125 MHz ^{13}C-NMR spectrum of compound **1** in CD_3OD.

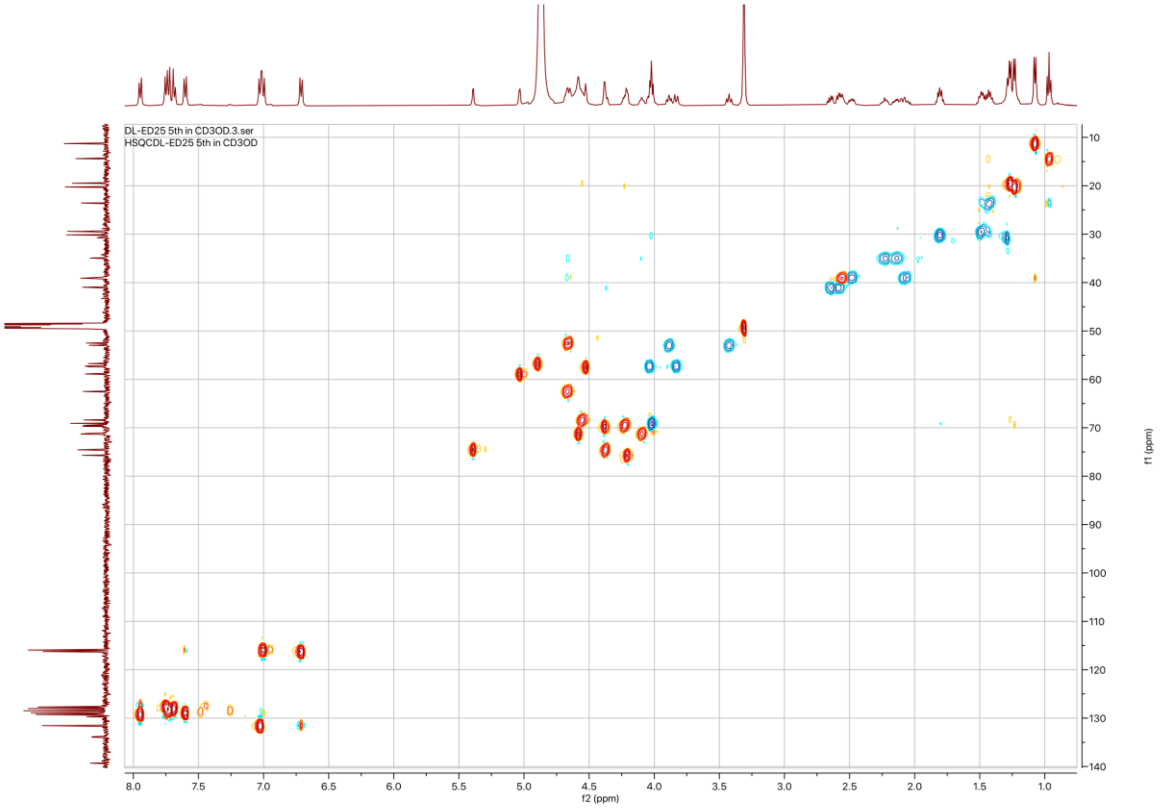

Figure S3. HSQC spectrum of compound **1** in CD_3OD.

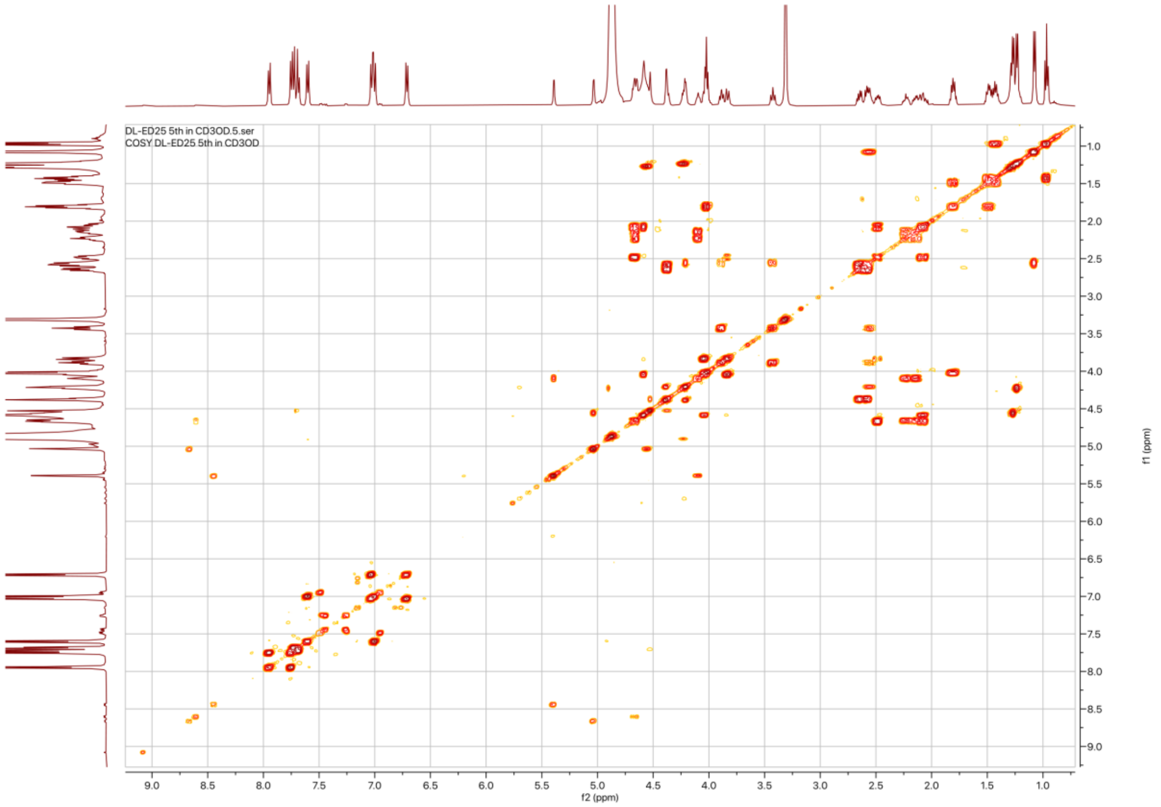

Figure S4. COSY spectrum of compound **1** in CD_3OD.

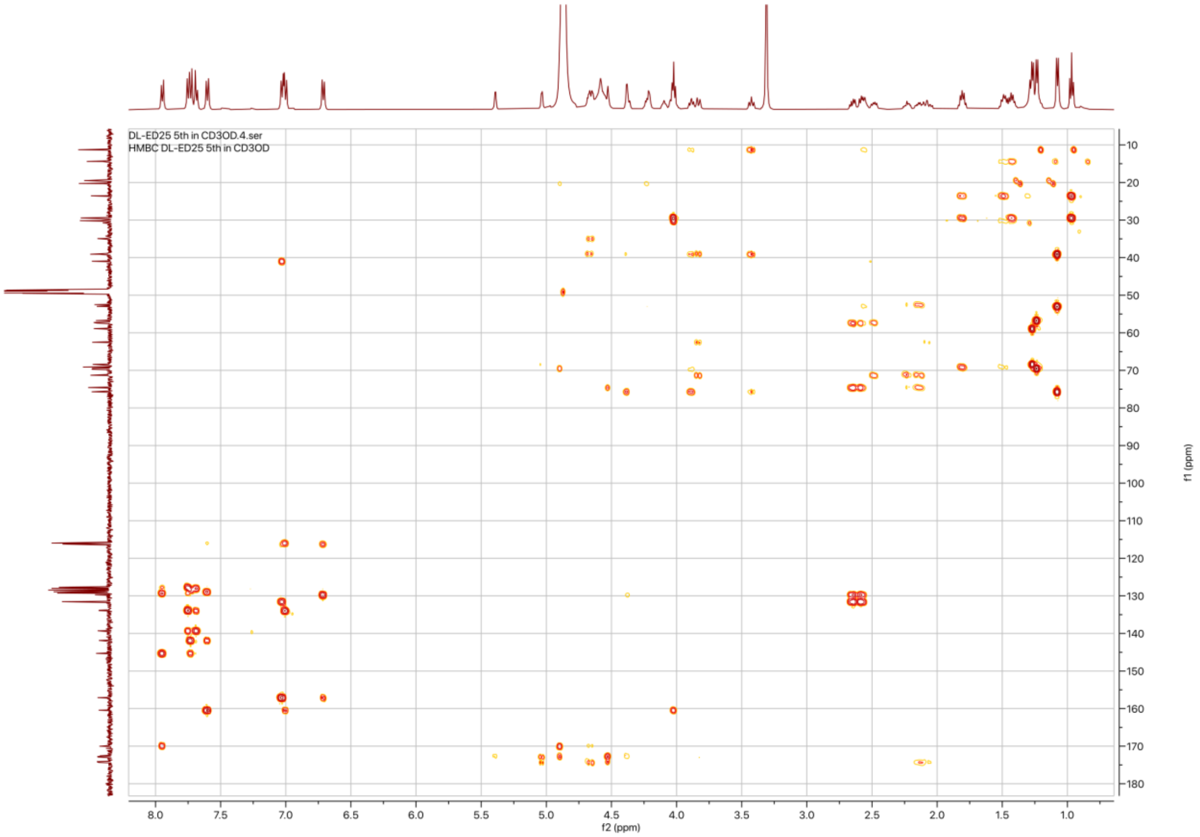

Figure S5. HMBC spectrum of compound **1** in CD_3OD.

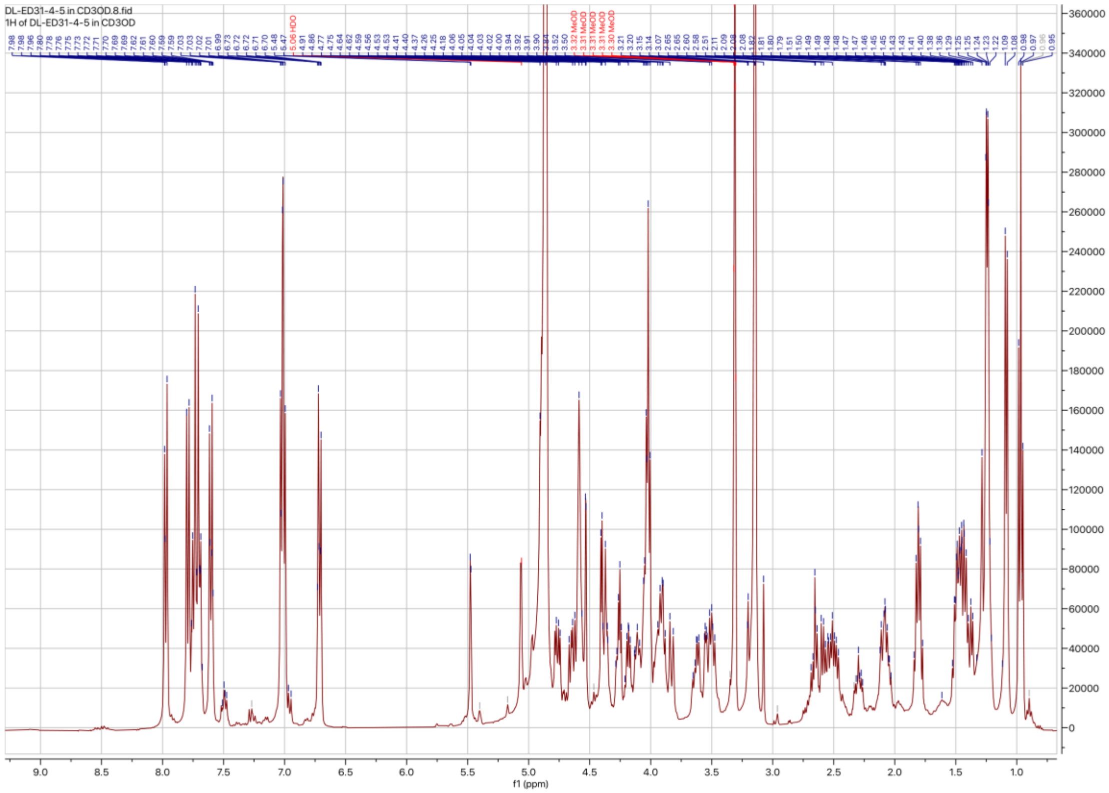

Figure S6. 400 MHz ^1H-NMR spectrum of compound **2** in CD_3OD.

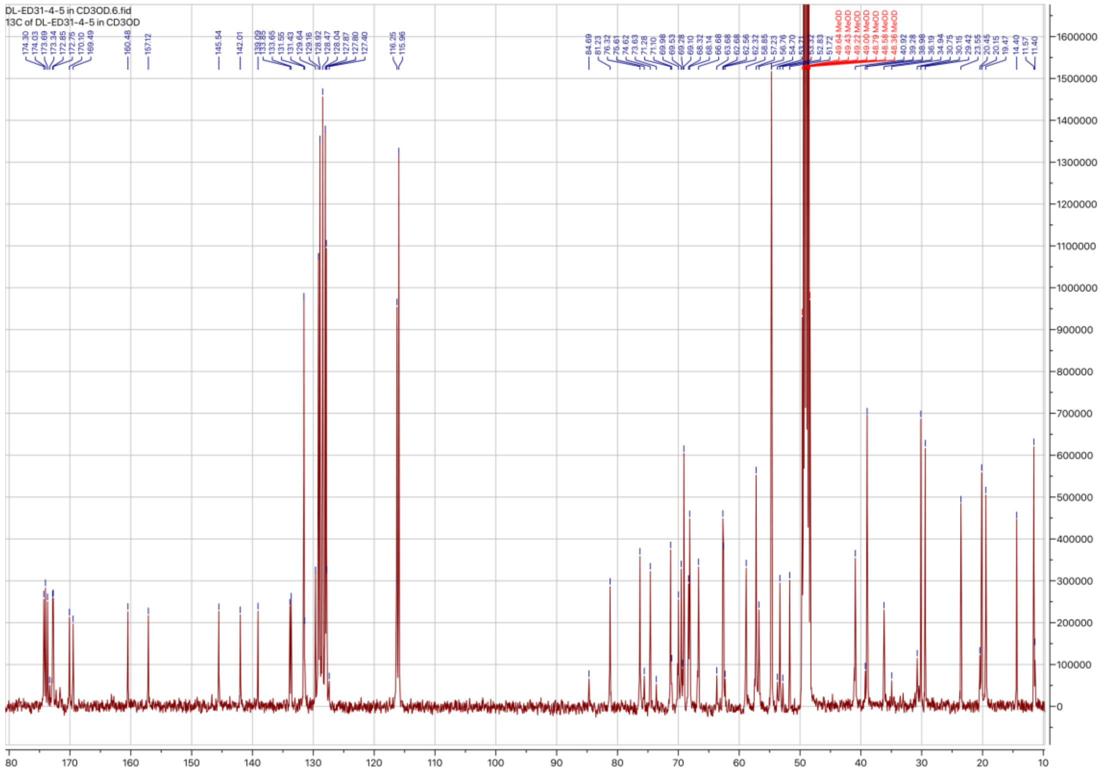

Figure S7. 100 MHz ^{13}C-NMR spectrum of compound **2** in CD_3OD.

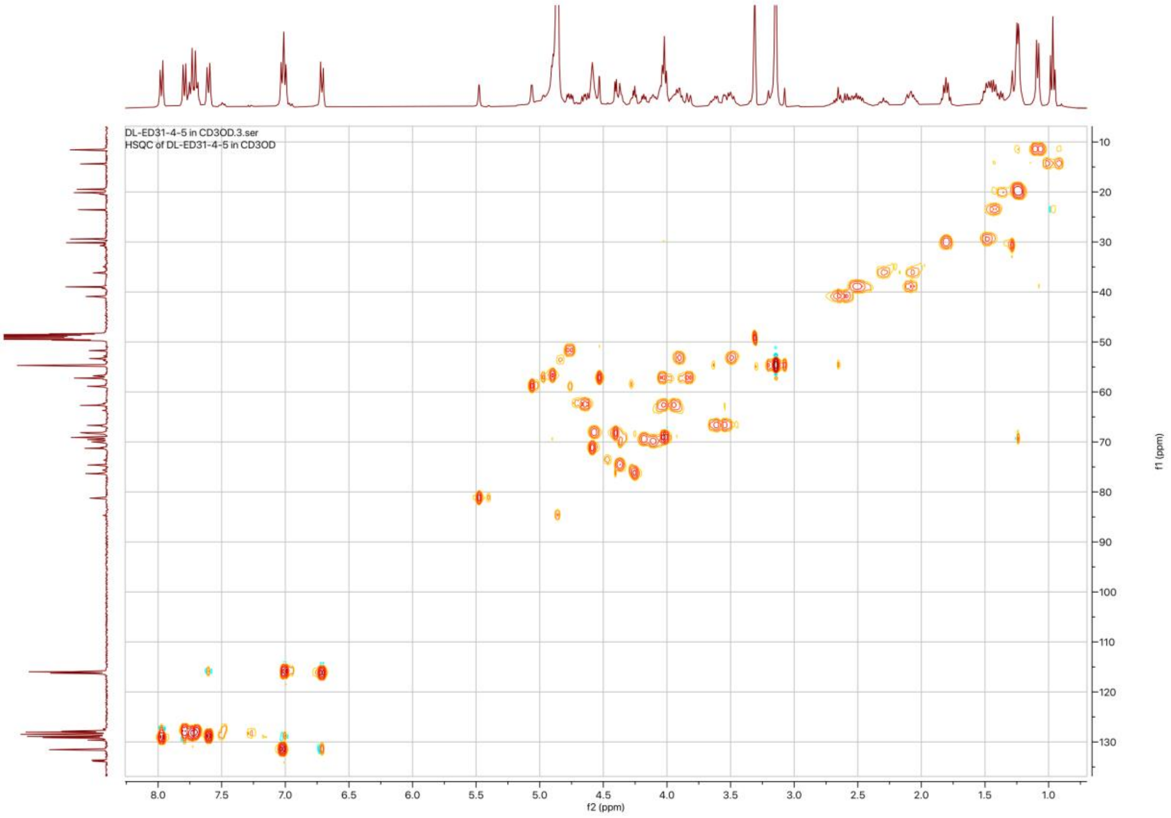

Figure S8. HSQC spectrum of compound **2** in CD_3OD

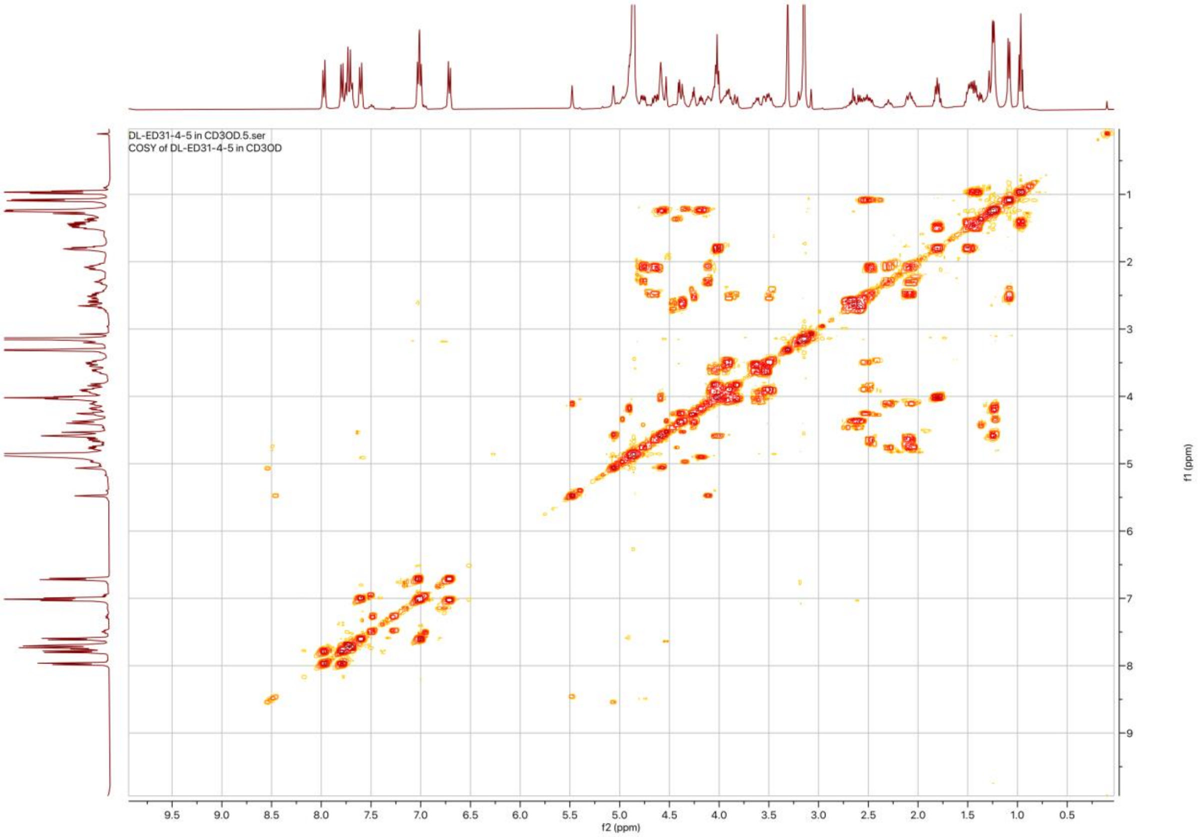

Figure S9. COSY spectrum of compound **2** in CD_3OD.

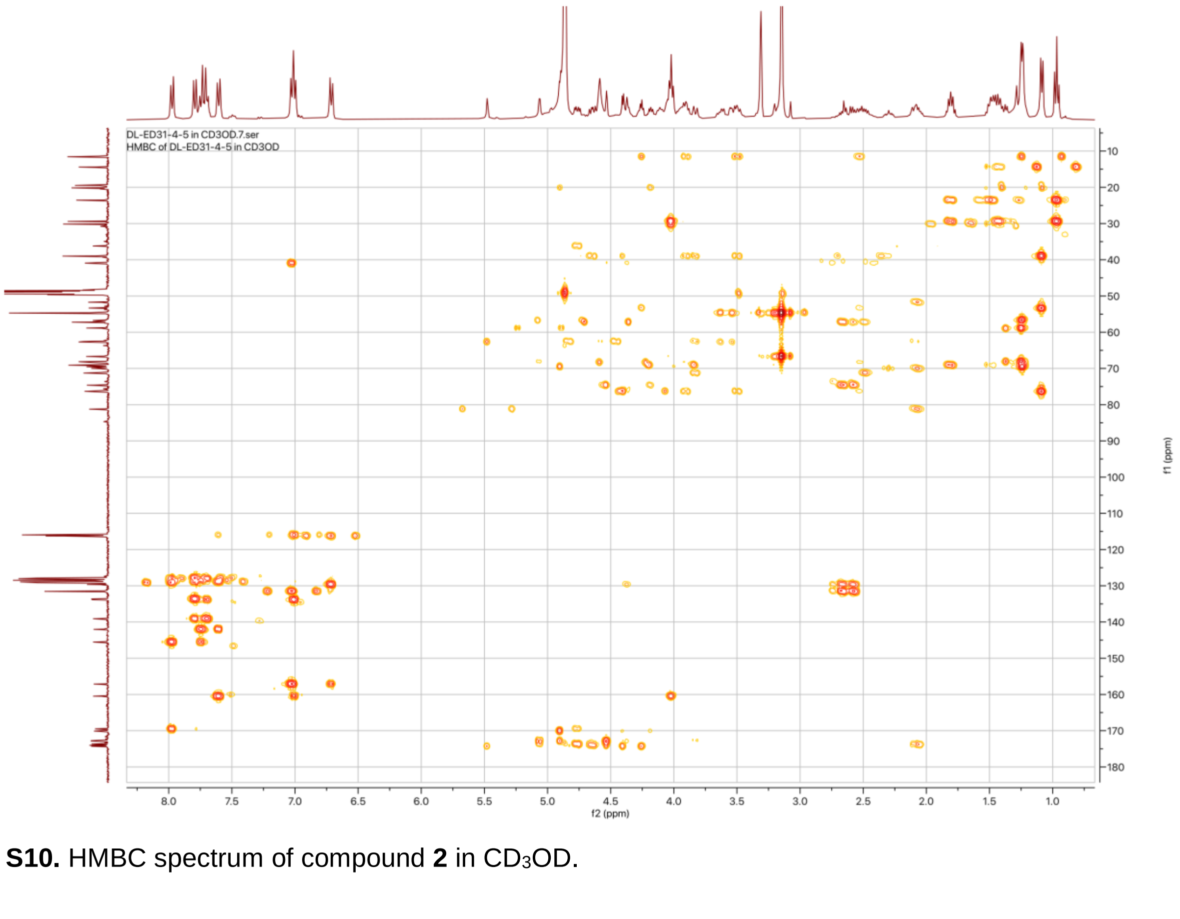

Figure S10. HMBC spectrum of compound **2** in CD_3OD.

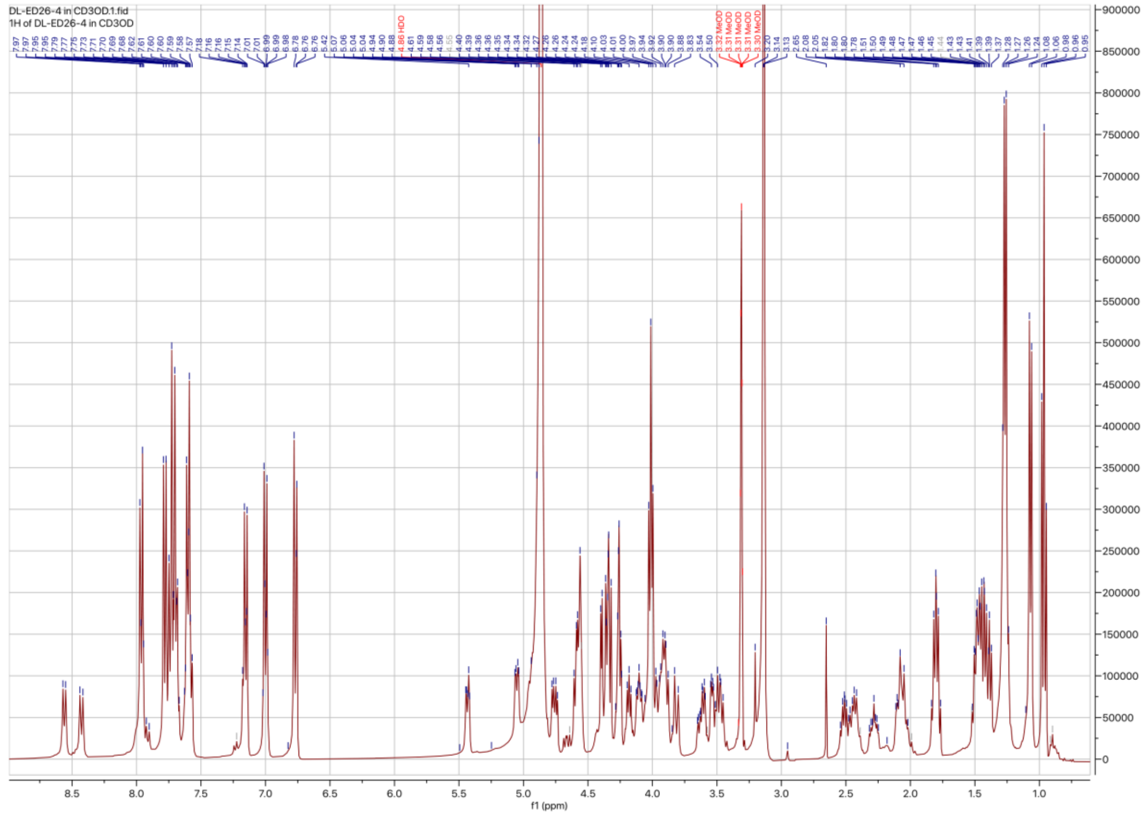

Figure S11. 400 MHz ^{1}H-NMR spectrum of **RZF** in CD_3OD.

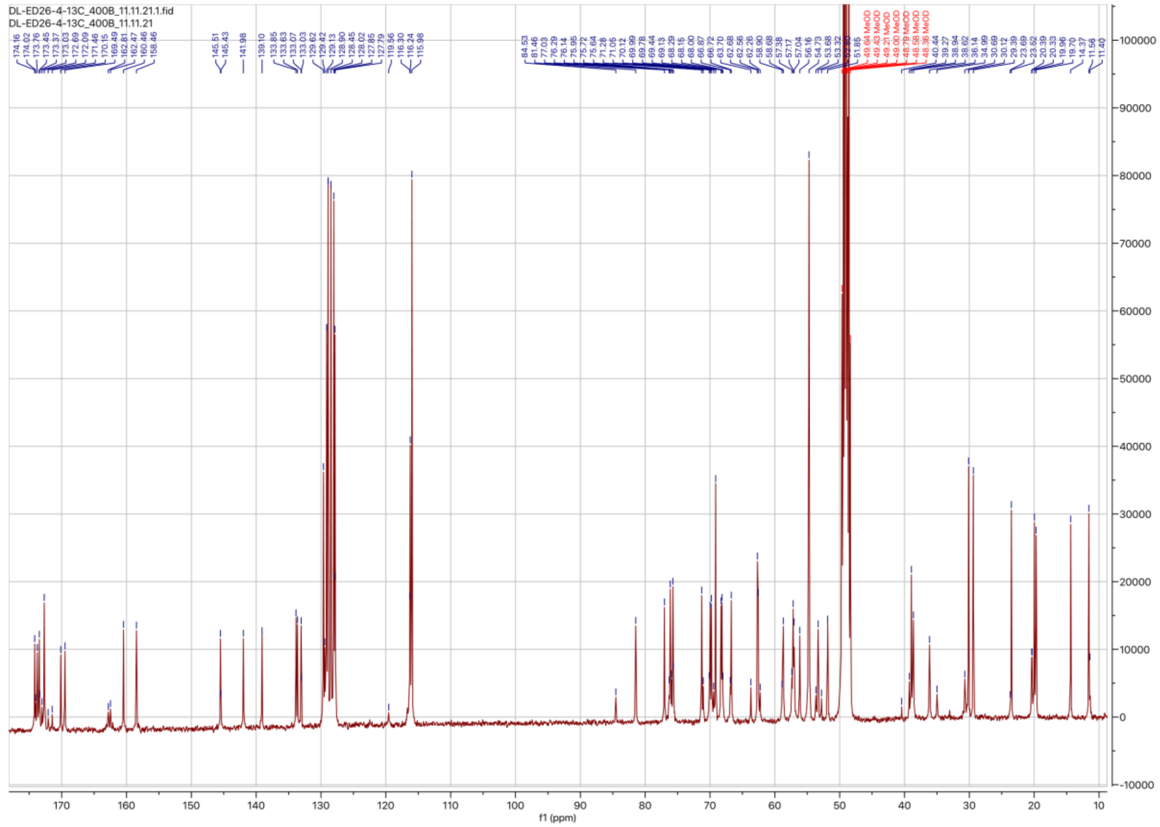

Figure S12. 100 MHz ^{13}C-NMR spectrum of **RZF** in CD_3OD.

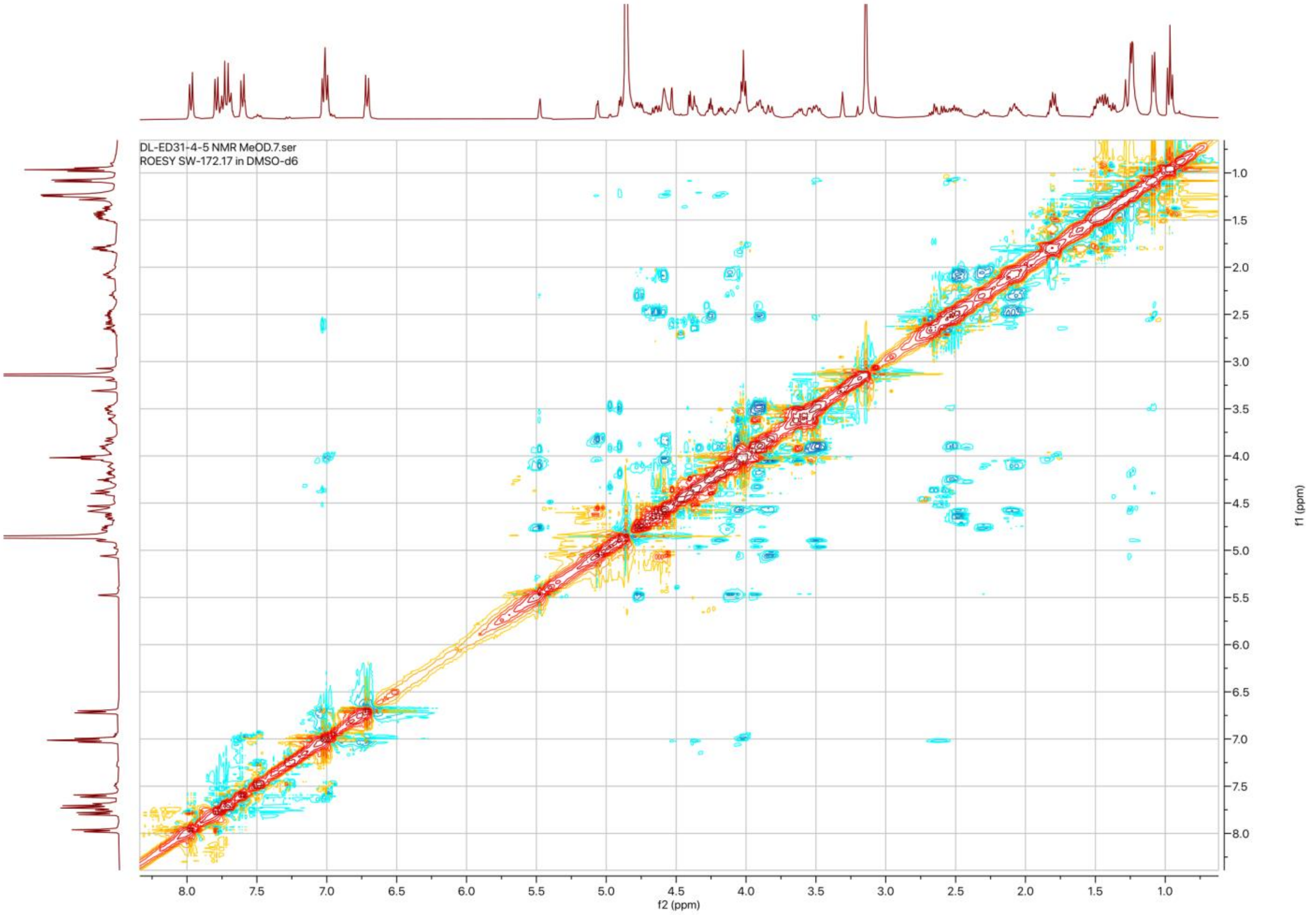

Figure S13. ROESY spectrum of compound **2** in CD_3OD.

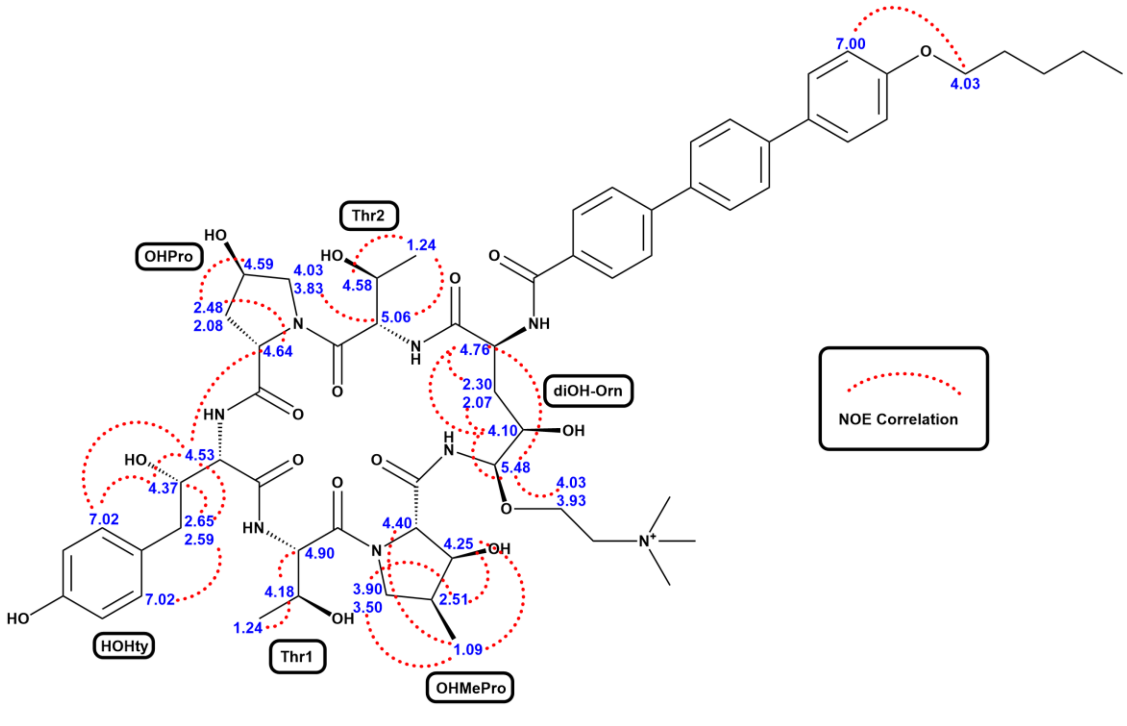

Figure S14. NOE correlations of compound **2** in CD_3OD.

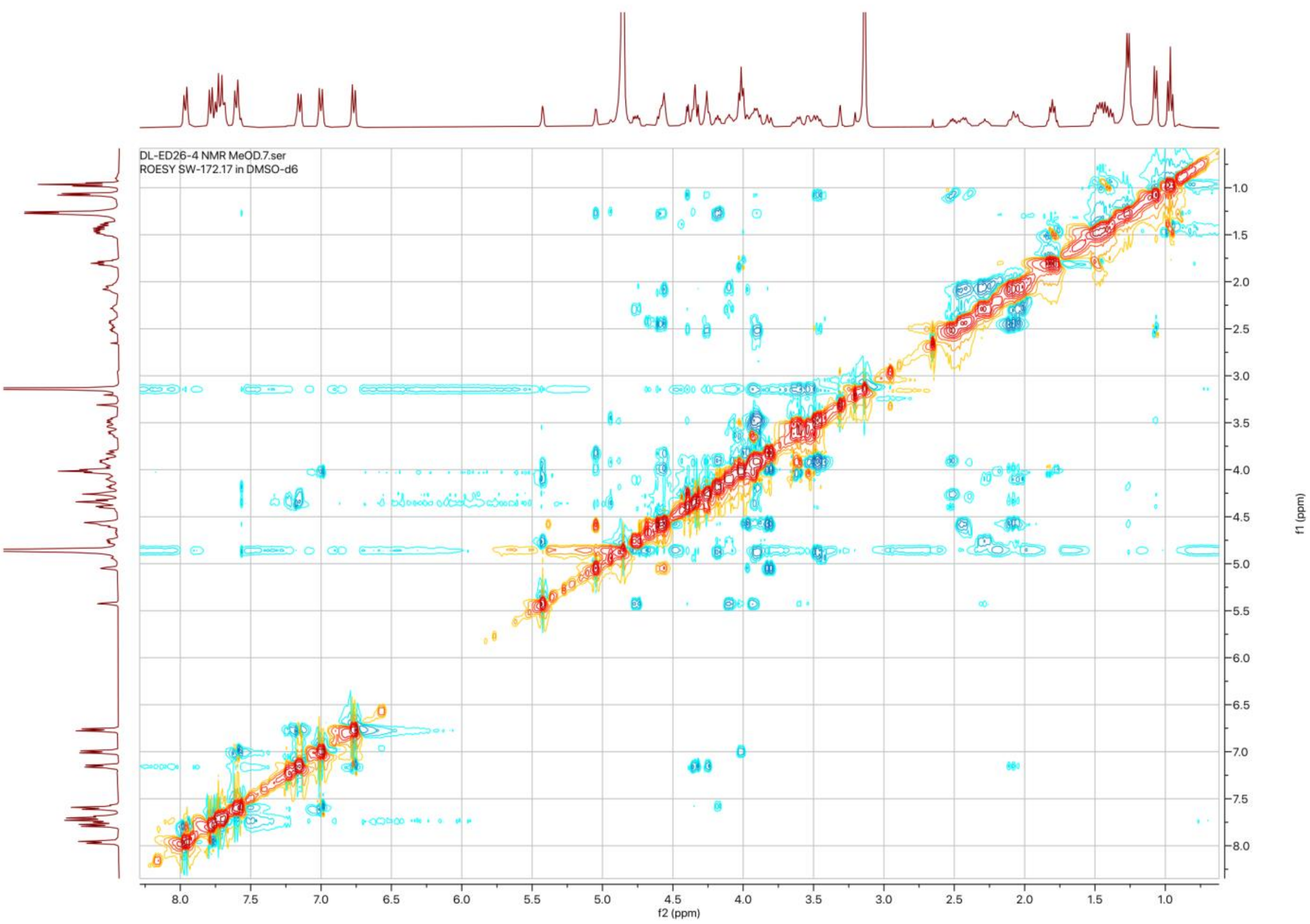

Figure S15. ROESY spectrum of **RZF** in CD$_3$OD.

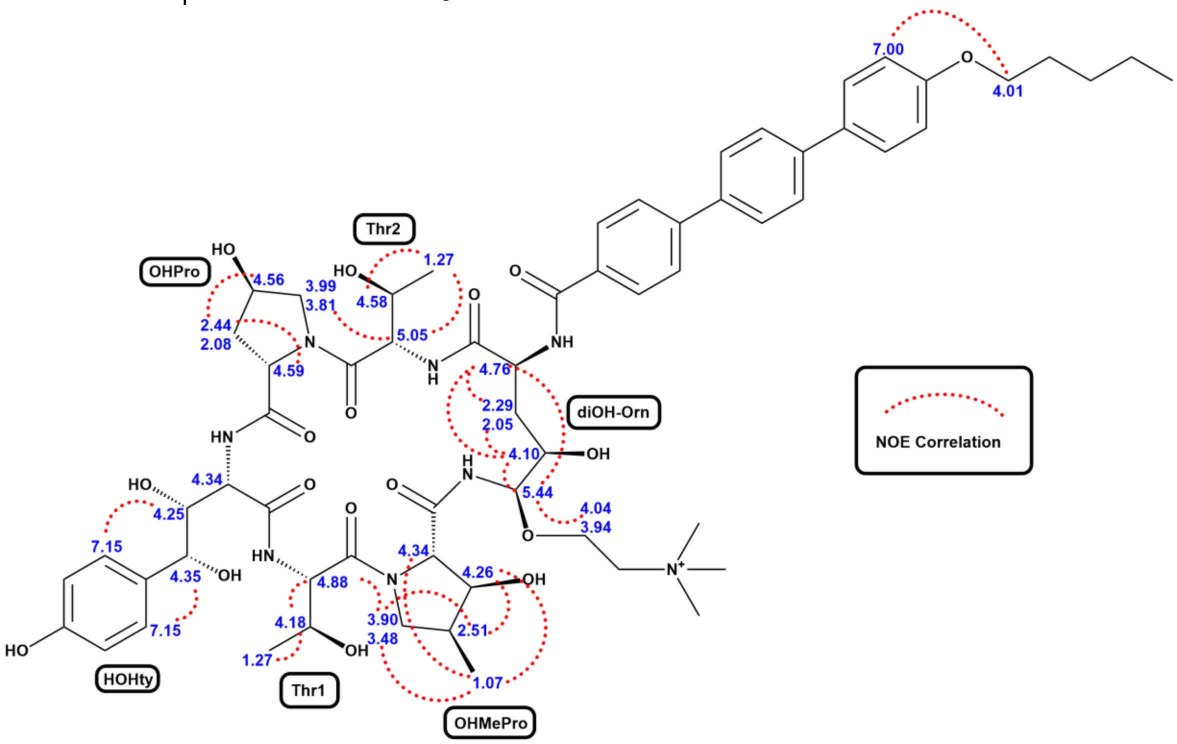

Figure S16. NOE correlations of **RZF** in CD$_3$OD.

1.3. Analytical HPLC chromatograms

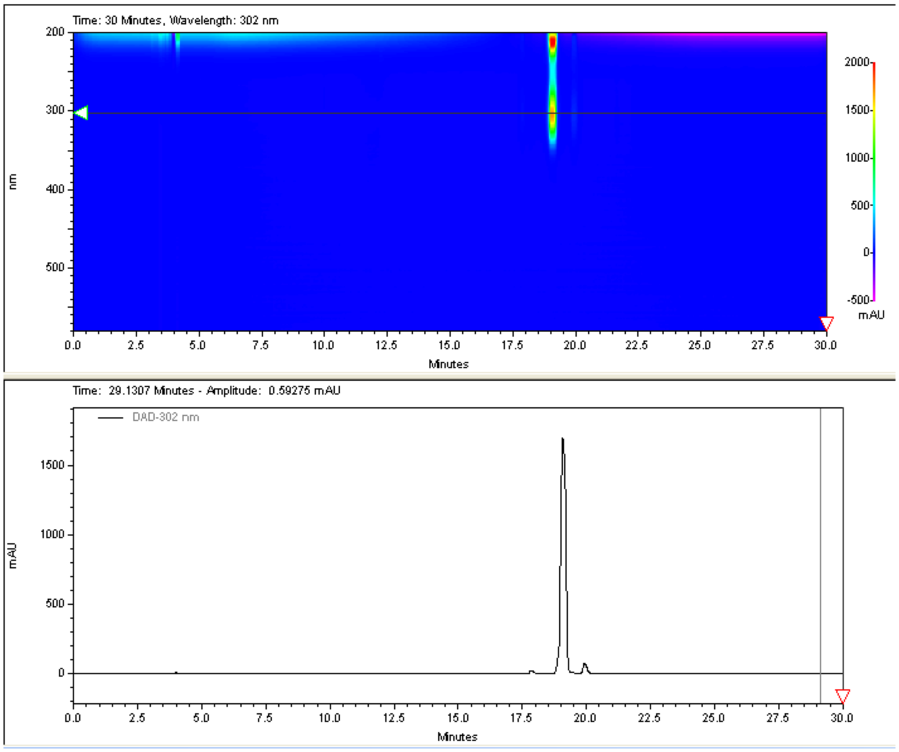

Figure S17. Analytic RF-HPLC chromatogram (diode array detector) of compound **1**. HPLC conditions: mobile phase: Acetonitrile in H_2O (containing 0.1% TFA), gradient from 10% to 90%; flow rate: 1 mL/min.

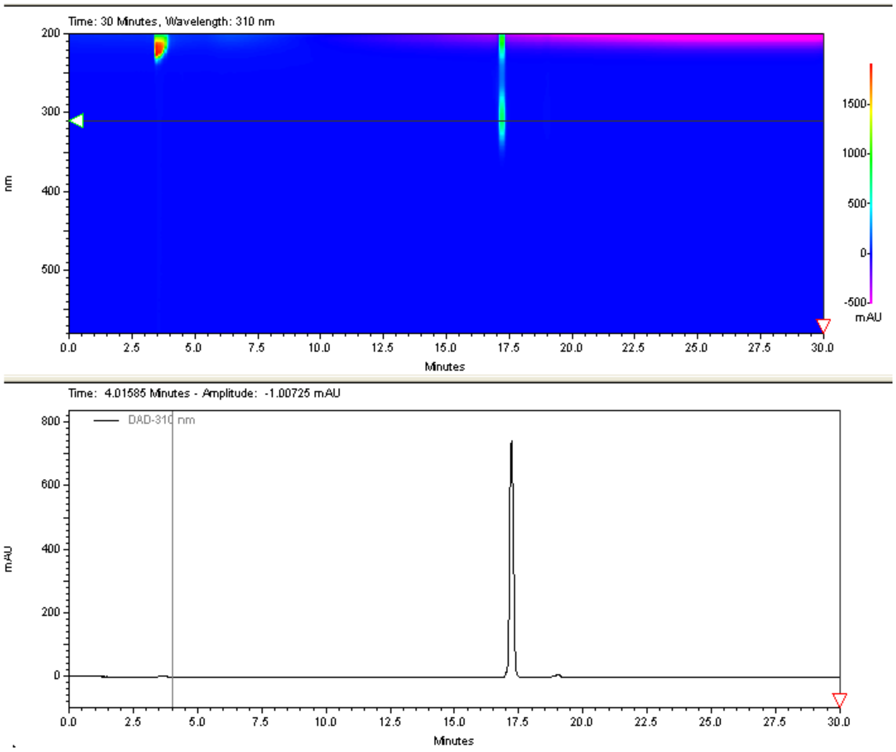

Figure S18. Analytic RF-HPLC chromatogram (diode array detector) of compound **2**. HPLC conditions: mobile phase: Acetonitrile in H_2O (containing 0.1% TFA), gradient from 10% to 90%; flow rate: 1 mL/min.

2. BIOLOGY

2.1. Yeast strains

Table S4. Strains Information.

	#	Species	Strain Name	Isogenic parental strain	Genotype[a,b,c]	Source
1	**1**	***C. albicans***	**Sc5314**	**WT**		David Perlin[1]
	**2**	*C. albicans*	DPL1015 (DP-A15)	Sc5314	*FKS1* HS1 S645S/P	David Perlin[1]
	**3**	*C. albicans*	DPL1016 (DP-A15-10)	Sc5314	*FKS1* HS1 S645P	David Perlin[1]
	**4**	*C. albicans*	T-2068 (DP-C42)	Sc5314	*FKS1* HS1 F641S	David Perlin[2]
2	**5**	*C. albicans*	T-2069 (DP-85)	clinical isolate	*FKS1* HS1 S645F	David Perlin[1]
	**6**	*C. albicans*	(DP-89)	clinical isolate	*FKS1* HS1 S645Y	David Perlin[1]
	**7**	*C. albicans*	T-2076 (DP-194)	clinical isolate	*FKS1* HS1 S645F; HS2 R1361R/H	David Perlin[1]
	**8**	*C. albicans*	T-2077 (DP-205)	clinical isolate	*FKS1* HS1 S645P	David Perlin[1]
3	**9**	***C. glabrata***	**EF1620**			Toni Gabaldon[3]
	**10**	*C. glabrata*	TGL00277	EF1620	*FKS1* HS1 F625Y, D632E	Toni Gabaldon[4]
	**11**	*C. glabrata*	TGL00275	EF1620	*FKS2* HS1 L662F, D666N	Toni Gabaldon[4]
4	**12**	***C. glabrata***	**CST78**			Toni Gabaldon[3]
	**13**	*C. glabrata*	TGL00265	CST78	*FKS1* W611*; *FKS2* HS1 F659Y	Toni Gabaldon[4]
	**14**	*C. glabrata*	TGL00264	CST78	*FKS2* HS1 R665G; HS2 R1378C; L1381F	Toni Gabaldon[4]
	**15**	*C. glabrata*	TGL00263	CST78	*FKS2* HS1 F659S	Toni Gabaldon[4]
	**16**	*C. glabrata*	TGL00065	CST78	*FKS1* HS1 L628I, D632N	Toni Gabaldon[4]
5	**17**	***C. glabrata***	**CST34**			Toni Gabaldon[3]
	**18**	*C. glabrata*	TGL00258	CST34	*FKS1* HS1 S629P	Toni Gabaldon[4]
	**19**	*C. glabrata*	TGL00256	CST34	*FKS1* HS1 D632G; *FKS2* HS1 F659L, D666Y	Toni Gabaldon[4]
	**20**	*C. glabrata*	TGL00107	CST34	*FKS1* HS1 S629P, P633Q	Toni Gabaldon[4]
	**21**	*C. glabrata*	TGL00056	CST34	*FKS1* E621K; *FKS2* HS1 F659-, D666Y	Toni Gabaldon[4]
6	**22**	***C. glabrata***	**M12**			Toni Gabaldon[3]
	**23**	*C. glabrata*	TGL00270	M12	*FKS2* HS1 F659C; HS2 R1378L	Toni Gabaldon[4]
	**24**	*C. glabrata*	TGL00269	M12	*FKS1* HS1 D632Y; *FKS2* HS1 L660S; HS2 R1378H	Toni Gabaldon[4]
	**25**	*C. glabrata*	TGL00268	M12	*FKS1* W705*; *FKS2* HS1 F659-; HS2 S1371S	Toni Gabaldon[4]
	**26**	*C. glabrata*	TGL00268	M12	*FKS1* HS1 F625V; *FKS2* W683*	Toni Gabaldon[4]
7	**27**	***C. glabrata***	**EB0911**			Toni Gabaldon[3]
	**28**	*C. glabrata*	TGL00261	EB0911	*FKS2* HS1 F659-	Toni Gabaldon[4]
	**29**	*C. glabrata*	TGL00109	EB0911	*FKS2* HS1 F659S; HS2 R1378S	Toni Gabaldon[4]
	**30**	*C. glabrata*	TGL00259	EB0911	*FKS2* HS1 S663P	Toni Gabaldon[4]

8	**31**	***C. glabrata***	**EF1237**			Toni Gabaldon[3]
	**32**	*C. glabrata*	TGL00273	EF1237	*FKS1* HS1 D632E; *FKS2* I670X-Frame Shift [a]	Toni Gabaldon[4]
	**33**	*C. glabrata*	TGL00272	EF1237	*FKS1* HS1 S629P, D632Y	Toni Gabaldon[4]
	**34**	*C. glabrata*	TGL00271	EF1237	*FKS1* HS1 D632N; *FKS2* I717N; HS2 R1378H	Toni Gabaldon[4]
	**35**	*C. glabrata*	TGL00073	EF1237	*FKS2* HS1 F659-, D666Y	Toni Gabaldon[4]
9	**36**	***C. glabrata***	**F15**			Toni Gabaldon[3]
	**37**	*C. glabrata*	TGL00281	F15	*FKS1* R643*; *FKS2* HS1 I661M, P667T	Toni Gabaldon[4]
10	**38**	***C. glabrata***	**CBS138 (ATCC 2001)**	**WT**		Toni Gabaldon[3]
	**39**	*C. glabrata*	TGL00133	CBS138	*FKS2* HS1 S663P; I1385T	Toni Gabaldon[4]
	**40**	*C. glabrata*	TGL00284	CBS138	*FKS2* HS1 P667H	Toni Gabaldon[4]
11	**41**	***C. glabrata***	**P35_2**			Toni Gabaldon[3]
	**42**	*C. glabrata*	TGL00287	P35_2	*FKS2* HS1 F659S, D666N	Toni Gabaldon[4]
12	**43**	***C. glabrata***	**SLL2glab**			Toni Gabaldon[3]
	**44**	*C. glabrata*	TGL00298	SLL2glab	*FKS2* HS2 R1378S	Toni Gabaldon[4]
	**45**	*C. glabrata*	TGL00295	SLL2glab	*FKS1* F625Y; FKS2 HS2 Y1379*	Toni Gabaldon[4]
13	**46**	***C. glabrata***	**BG2**	**WT**		Toni Gabaldon[3]
	**47**	*C. glabrata*	TGL00294	BG2	*FKS2* HS1 L662W; HS2 R1378C	Toni Gabaldon[4]
	**48**	*C. glabrata*	TGL00293	BG2	*FKS2* A651V HS1 F659-	Toni Gabaldon[4]
	**49**	*C. glabrata*	TGL00291	BG2	*FKS2* S654Y HS1 F659-	Toni Gabaldon[4]
	**50**	*C. glabrata*	TGL00091	BG2	*FKS2* A651V HS1 S663P	Toni Gabaldon[4]

[a] Frame shift is marked by X; [b] Dash following the amino acid number indicates a deletion; [c] Asterisk indicates a premature termination codon.

2.2. Antifungal activity

Table S5. Minimal Inhibitory Concentration (MIC) values.

				MIC [μg/mL]					
	**#**	Species	Strain Name	**CSF**	**MCF**	**ANF**	**1**	**RZF**	**2**
1	**1**	***C. albicans***	**Sc5314**	0.015	0.031	0.004	0.008	0.015	0.015
	**2**	*C. albicans*	DPL1015 (DP-A15)	1	1	0.063	0.031	0.25	0.062
	**3**	*C. albicans*	DPL1016 (DP-A15-10)	>64	16	4	1	4	2
	**4**	*C. albicans*	T-2068 (DP-C42)	32	4	2	0.25	2	0.5
2	**5**	*C. albicans*	T-2069 (DP-85)	16	2	2	0.5	8	0.5
	**6**	*C. albicans*	(DP-89)	>64	4	2	1	8	1
	**7**	*C. albicans*	T-2076 (DP-194)	1	1	0.5	0.5	1	1
	**8**	*C. albicans*	T-2077 (DP-205)	16	2	0.5	0.125	1	0.25
3	**9**	***C. glabrata***	**EF1620**	0.031	0.015	0.008	0.004	0.015	0.015
	**10**	*C. glabrata*	TGL00277	>64	8	>64	8	64	16
	**11**	*C. glabrata*	TGL00275	32	2	4	8	16	8
4	**12**	***C. glabrata***	**CST78**	0.015	0.004	0.004	0.002	0.008	0.004
	**13**	*C. glabrata*	TGL00265	>64	1	4	1	16	2
	**14**	*C. glabrata*	TGL00264	4	>64	32	>64	32	32
	**15**	*C. glabrata*	TGL00263	64	0.125	2	1	32	2
	**16**	*C. glabrata*	TGL00065	>64	32	8	16	>64	8
5	**17**	***C. glabrata***	**CST34**	0.015	0.002	0.008	0.002	0.008	0.004
	**18**	*C. glabrata*	TGL00258	>64	>64	>64	32	>64	8
	**19**	*C. glabrata*	TGL00256	16	0.125	2	0.25	4	0.25
	**20**	*C. glabrata*	TGL00107	>64	>64	>64	>64	>64	16
	**21**	*C. glabrata*	TGL00056	>64	>64	>64	>64	>64	>64
6	**22**	***C. glabrata***	**M12**	0.015	0.002	0.002	0.002	0.008	0.002
	**23**	*C. glabrata*	TGL00270	>64	16	4	1	>64	4
	**24**	*C. glabrata*	TGL00269	16	0.125	0.25	0.063	1	0.063
	**25**	*C. glabrata*	TGL00268	>64	>64	>64	>64	>64	>64
	**26**	*C. glabrata*	TGL00268	>64	>64	>64	>64	>64	>64
7	**27**	***C. glabrata***	**EB0911**	0.031	0.008	0.004	0.002	0.015	0.004
	**28**	*C. glabrata*	TGL00261	>64	4	2	0.25	16	1
	**29**	*C. glabrata*	TGL00109	64	>64	>64	64	64	64
	**30**	*C. glabrata*	TGL00259	>64	32	16	4	>64	8

8	**31**	***C. glabrata***	**EF1237**	0.008	0.004	0.002	0.001	0.008	0.002
	**32**	*C. glabrata*	TGL00273	8	0.5	4	1	8	1
	**33**	*C. glabrata*	TGL00272	>64	8	4	2	64	4
	**34**	*C. glabrata*	TGL00271	>64	>64	>64	64	>64	32
	**35**	*C. glabrata*	TGL00073	>64	>64	>64	>64	>64	>64
9	**36**	***C. glabrata***	**F15**	0.015	0.002	0.004	0.002	0.015	0.004
	**37**	*C. glabrata*	TGL00281	16	2	2	0.5	4	0.5
10	**38**	***C. glabrata***	**CBS138 (ATCC 2001)**	0.015	0.008	0.008	0.004	0.031	0.008
	**39**	*C. glabrata*	TGL00133	>64	>64	>64	>64	>64	>64
	**40**	*C. glabrata*	TGL00284	>64	>64	>64	>64	>64	64
11	**41**	***C. glabrata***	**P35_2**	0.031	0.008	0.004	0.002	0.015	0.004
	**42**	*C. glabrata*	TGL00287	16	0.125	8	0.5	4	0.5
12	**43**	***C. glabrata***	**SLL2glab**	0.031	0.002	0.008	0.002	0.008	0.008
	**44**	*C. glabrata*	TGL00298	>64	>64	>64	>64	>64	>64
	**45**	*C. glabrata*	TGL00295	>64	1	8	0.25	32	0.5
13	**46**	***C. glabrata***	**BG2**	0.015	0.004	0.004	0.001	0.015	0.008
	**47**	*C. glabrata*	TGL00294	>64	8	8	4	>64	64
	**48**	*C. glabrata*	TGL00293	>64	>64	64	16	>64	32
	**49**	*C. glabrata*	TGL00291	>64	>64	>64	>64	>64	>64
	**50**	*C. glabrata*	TGL00091	>64	>64	8	16	16	32

2.3. Mammalian cell viability.

Table S6. Effects of ANF, RZF, and dehydroxylated derivatives **1** and **2** on the viability of human-derived cell lines.

	IC$_{50}$ [μM]					
**Cell line**[a]	**ANF**	**1**	**RZF**	**2**	**Staurosporine**	**Tamoxifen**
**HCT-116**	>40	>40	>40	34.13 ± 11.50	0.54 ± 1.10	--
**UACC-62**	34.51 ± 4.15	>40	>40	29.19 ± 5.2	0.62 ± 0.49	--
**Hs 578T**	>40	>40	>40	>40	0.80 ± 0.58	--
**SF-295**	19.28 ± 1.97	17.49 ± 1.57	>40	32.35 ± 6.7	0.34 ± 0.25	--
**THLE-2**	40.6 ± 2.90	36.4 ± 2.75	76.6 ± 4.80	55.9 ± 3.0	--	19.6 6 ± 1.0

[a]HCT-116 (human colorectal carcinoma cell line); UACC-62 (human melanoma cell line); Hs 578T (human mammary gland carcinoma cell line); SF-295 (human glioblastoma cell line);THLE-2 (transformed human liver epithelial cells).

3. DFT CALCULATIONS

DFT calculations were performed using Gaussian 09.2.[5] Geometry optimization of all the molecules were carried out using the BP86-D3 method[6,7] with Ahlrichs' def2-SVP basis set[8] implemented in the Gaussian 09 software. Conductor-like polarizable continuum model (CPCM)[9,10] was used to account for the effect of H_2O. Thermal energy corrections were extracted from the results of frequency analysis performed at the same level of theory. Frequency analysis of all the molecules and intermediates contained no imaginary frequency showing that these are energy minima.

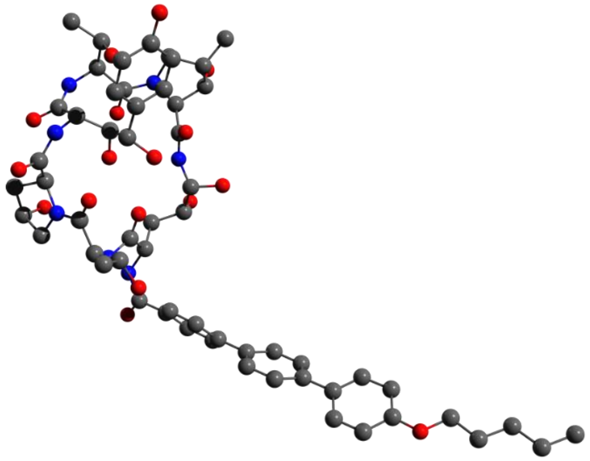

ANF:

O	0.03490	1.61985	2.67988
C	0.98797	0.60039	2.40656
N	2.32524	1.00768	2.83789
C	0.56652	-0.63500	3.22538
C	2.98516	2.00506	2.21062
O	2.60617	2.46810	1.11313
O	0.87827	-0.43004	4.60809
C	1.23742	-1.94714	2.78064
C	0.46150	-2.70000	1.66274
N	0.29037	-4.12501	1.94120
C	1.04221	-2.43433	0.26106
O	1.45195	-1.30376	-0.04323
N	1.01226	-3.47490	-0.60544
C	1.32885	-3.34325	-2.02778
C	2.74194	-2.71455	-2.22134
C	0.23536	-2.52636	-2.77653
C	0.36761	-2.64542	-4.29462
N	3.77740	-3.40423	-1.67560
O	2.89110	-1.64732	-2.83288
C	5.13367	-2.81754	-1.51642
C	5.99557	-4.06555	-1.27760
C	5.07036	-4.95409	-0.43741
C	3.71948	-4.77611	-1.13315
O	4.91465	-4.45266	0.89552
N	5.33954	2.81830	2.11773
C	5.52375	4.21835	1.68826
C	4.23090	4.92434	2.14123

C	3.80224	4.10889	3.38601
C	6.06276	1.68374	1.85937
O	4.47276	4.48188	4.58188
C	7.43625	1.71248	1.10930
O	5.69040	0.59655	2.31970
C	5.60487	-2.01613	-2.73761
N	5.94971	-0.69612	-2.57197
O	5.72717	-2.56616	-3.84119
C	6.05405	0.16304	-1.39275
C	7.82500	2.87430	0.16737
N	7.70304	0.38453	0.55430
C	7.12258	-0.40191	-0.41296
O	7.54499	-1.55769	-0.55136
C	4.69414	0.58998	-0.76396
C	3.72527	1.18787	-1.83815
C	4.23655	2.52022	-2.36876
O	2.40942	1.27468	-1.30439
C	5.00178	2.59412	-3.54868
C	5.55151	3.81040	-3.99197
C	5.32178	4.99553	-3.25812
C	4.53473	4.93877	-2.08443
C	4.00714	3.71548	-1.65251
O	5.81808	6.21142	-3.63155
C	9.21723	2.68111	-0.44038
H	5.10243	-2.17885	-0.61759
C	4.37924	6.42614	2.37208
C	-3.27365	-4.20532	2.76476
C	-4.51540	-3.65809	2.42117
C	-4.72560	-3.05221	1.15485
C	-3.64005	-3.03606	0.24449
C	-2.38979	-3.56568	0.59205
C	-2.18565	-4.14328	1.86609
C	-6.83221	-1.80544	1.78234
C	-8.06077	-1.21995	1.45227
C	-8.55833	-1.24616	0.12468
C	-7.76316	-1.89465	-0.85411
C	-6.53295	-2.47892	-0.52502
C	-6.03592	-2.44767	0.80164
C	-10.30452	0.55456	0.43550
C	-11.52741	1.16749	0.11779
C	-12.35065	0.61151	-0.88899
C	-11.91858	-0.55795	-1.56114
C	-10.69678	-1.15278	-1.23491
C	-9.85607	-0.61381	-0.22479
O	-13.55222	1.11821	-1.27648
C	-0.88505	-4.77510	2.28734
O	-0.85918	-5.84847	2.89991
C	-14.05586	2.29491	-0.62467
C	-15.40927	2.63672	-1.23528
C	-16.03688	3.88390	-0.59773
C	-17.40415	4.25337	-1.19331
C	-18.02545	5.49926	-0.55022
H	0.24153	2.36179	2.07572
H	1.03208	0.34728	1.32636
H	2.58355	0.74230	3.79632
H	-0.53708	-0.72642	3.08855
H	0.38667	0.36973	4.88883
H	1.25471	-2.59773	3.67684
H	2.28917	-1.76319	2.47821
H	-0.55877	-2.26442	1.60916
H	1.12803	-4.62453	2.26456
H	0.60951	-4.35006	-0.25798

H	1.34202	-4.37584	-2.43885
H	1.34751	-2.25907	-4.63364
H	-0.43842	-2.06536	-4.78696
H	0.27661	-3.70702	-4.61328
H	6.94639	-3.81376	-0.77447
H	6.21752	-4.55305	-2.25093
H	5.38708	-6.02336	-0.43369
H	2.90113	-4.89965	-0.40007
H	3.58618	-5.51810	-1.95011
H	5.80885	-4.36200	1.27857
H	5.65733	4.27341	0.59196
H	6.41584	4.66749	2.18187
H	3.45833	4.73412	1.36793
H	2.71933	4.21877	3.60051
H	5.43557	4.38394	4.43850
H	8.15288	1.80226	1.95423
H	6.33977	-0.30286	-3.43292
H	6.47873	1.12341	-1.74996
H	8.37112	-0.17438	1.09208
H	3.66351	0.45113	-2.66359
H	2.44400	1.72388	-0.41887
H	5.18119	1.68052	-4.13876
H	6.15152	3.84668	-4.91654
H	4.36144	5.86565	-1.51558
H	3.42465	3.67721	-0.71997
H	6.33974	6.10643	-4.45185
H	9.99206	2.59990	0.34947
H	9.47118	3.55201	-1.07905
H	9.24899	1.76392	-1.06112
H	5.09202	6.63250	3.19650
H	3.40493	6.88205	2.64283
H	4.75006	6.93092	1.45638
H	-3.13046	-4.68982	3.74295
H	-5.34712	-3.72152	3.13987
H	-3.75428	-2.57592	-0.74918
H	-1.58674	-3.51649	-0.15946
H	-6.47487	-1.74527	2.82228
H	-8.65488	-0.74386	2.24793
H	-8.10452	-1.92515	-1.90072
H	-5.95214	-2.98208	-1.31392
H	-9.67022	1.02228	1.20488
H	-11.82242	2.08123	0.65155
H	-12.57160	-0.98862	-2.33566
H	-10.39938	-2.07488	-1.75867
H	-14.15407	2.10940	0.47104
H	-13.33937	3.13956	-0.75822
H	-16.08680	1.76266	-1.11496
H	-15.28042	2.78892	-2.32968
H	-15.34229	4.74824	-0.70995
H	-16.14566	3.72471	0.49998
H	-18.09573	3.38740	-1.08197
H	-17.29296	4.41124	-2.29025
H	-18.17809	5.35449	0.54105
H	-19.01121	5.74398	-0.99774
H	-17.36897	6.38702	-0.67694
O	4.08273	-0.46350	-0.04980
H	3.12375	-0.51339	-0.29692
H	4.92658	1.42669	-0.06675
H	7.84864	3.79819	0.78977
O	6.81885	2.97273	-0.83415
H	7.01164	3.71306	-1.44313
C	4.15090	2.65178	2.96954

H	4.39898	2.04801	3.86502
O	-1.07142	-2.90856	-2.33505
H	-1.24917	-3.81160	-2.66800
H	0.35814	-1.46960	-2.46355

Sum of electronic and zero-point Energies= -3912.553574
Sum of electronic and thermal Energies= -3912.472922
Sum of electronic and thermal Enthalpies= -3912.471978
Sum of electronic and thermal Free Energies= -3912.677012

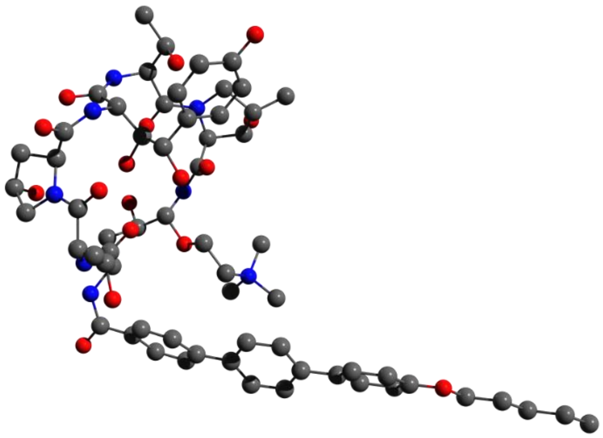

RZF:

O	-0.15768	-0.99501	-2.16647
C	-1.52397	-0.60270	-2.29175
N	-1.69842	0.67107	-2.98601
C	-2.20189	-1.74724	-3.09744
C	-1.79509	1.84501	-2.30949
O	-1.39748	2.00200	-1.13933
O	-3.24930	-1.18007	-3.88323
C	-2.77456	-2.86332	-2.20114
C	-1.74325	-3.56997	-1.26777
N	-1.70961	-5.01930	-1.46082
C	-2.01359	-3.13361	0.17780
O	-1.91268	-1.93250	0.48480
N	-2.39314	-4.07997	1.06312
C	-2.74495	-3.75009	2.44571
C	-3.76286	-2.56349	2.46599
C	-1.48426	-3.42254	3.29691
C	-1.79328	-3.41206	4.79358
N	-4.92477	-2.76128	1.78877
O	-3.49926	-1.50240	3.05035
C	-5.85576	-1.64831	1.45173
C	-7.13444	-2.40658	1.06743
C	-6.57808	-3.63919	0.34653
C	-5.41338	-4.04633	1.24864
O	-6.01041	-3.30421	-0.92696
N	-3.44074	3.67161	-2.25854
C	-3.02063	4.97791	-1.71387
C	-1.50317	5.01971	-1.97881
C	-1.34797	4.18611	-3.27389
C	-4.54974	2.90204	-2.02979
O	-1.60483	4.91502	-4.46680
C	-5.83759	3.45066	-1.32945

O	-4.60598	1.75619	-2.49917
C	-6.08365	-0.65805	2.59996
N	-5.79373	0.67303	2.39775
O	-6.56496	-1.03967	3.67537
C	-5.41238	1.45028	1.21677
C	-5.77577	4.64803	-0.35646
N	-6.64393	2.32437	-0.85229
C	-6.51860	1.36666	0.12863
O	-7.36890	0.46816	0.17447
C	-3.96316	1.22303	0.69596
C	-2.89076	1.38782	1.82102
C	-2.82459	2.82054	2.33229
O	-1.63046	0.92759	1.34391
C	-3.58582	3.24717	3.43713
C	-3.58940	4.59252	3.84754
C	-2.79968	5.54163	3.16024
C	-2.00840	5.11874	2.06691
C	-2.02933	3.77769	1.66564
O	-2.75018	6.86084	3.50732
C	-7.15257	5.00508	0.20956
H	-5.43912	-1.12695	0.57348
C	-0.90658	6.42311	-2.04093
C	1.81980	-5.14687	-2.16259
C	3.04428	-4.58107	-1.78343
C	3.23950	-4.04736	-0.48150
C	2.15968	-4.14480	0.43259
C	0.92509	-4.68132	0.04857
C	0.73395	-5.16909	-1.26265
C	5.70863	-3.56027	-0.81761
C	6.85859	-2.81266	-0.53556
C	6.85857	-1.80029	0.45793
C	5.64850	-1.58875	1.16586
C	4.49986	-2.33771	0.88614
C	4.49369	-3.33658	-0.12122
C	8.99692	-0.66508	-0.27165
C	10.15065	0.09185	-0.01060
C	10.41398	0.53890	1.30543
C	9.49835	0.21751	2.33739
C	8.35328	-0.53238	2.05938
C	8.07268	-0.99767	0.74685
O	11.49950	1.26825	1.67551
C	-0.57495	-5.77301	-1.70307
O	-0.63346	-6.88595	-2.23549
C	12.49059	1.60602	0.69158
C	13.60250	2.37618	1.39293
C	14.72685	2.79415	0.43536
C	15.86086	3.56285	1.13116
C	16.98400	3.98042	0.17405
H	-1.97450	-0.49948	-1.28581
H	-2.23919	0.58780	-3.85749
H	-1.40343	-2.17049	-3.75289
H	-3.46563	-1.81223	-4.59483
H	-3.21471	-3.62617	-2.87630
H	-3.62036	-2.44795	-1.61568
H	-0.73379	-3.17088	-1.49686
H	-2.57658	-5.47148	-1.77453
H	-2.34756	-5.05844	0.76399
H	-3.23601	-4.65335	2.86745
H	-2.56273	-2.65152	5.02724
H	-0.87299	-3.17997	5.36599
H	-2.16728	-4.40610	5.12332
H	-7.80107	-1.78701	0.44164

H	-7.67872	-2.71328	1.98610
H	-7.32652	-4.45961	0.24997
H	-4.64215	-4.56932	0.65510
H	-5.75350	-4.71606	2.06800
H	-6.68503	-2.81041	-1.43251
H	-3.24437	5.03551	-0.63282
H	-3.54329	5.81149	-2.23725
H	-1.01336	4.43199	-1.17504
H	-0.31988	3.78403	-3.38365
H	-2.50448	5.29493	-4.40864
H	-6.41274	3.84273	-2.19631
H	-6.08109	1.23499	3.20409
H	-5.40044	2.51029	1.54113
H	-7.41445	2.06783	-1.47562
H	-3.18120	0.70282	2.64439
H	-1.50575	1.27072	0.42200
H	-4.19833	2.51717	3.99086
H	-4.19589	4.90936	4.71220
H	-1.39387	5.86269	1.53651
H	-1.43305	3.46240	0.79687
H	-3.33818	7.01358	4.27359
H	-7.86978	5.24319	-0.60236
H	-7.07020	5.89724	0.86387
H	-7.56116	4.16442	0.80456
H	-1.33401	6.99893	-2.88713
H	0.19263	6.37767	-2.18214
H	-1.10819	6.98095	-1.10343
H	1.68752	-5.54602	-3.18002
H	3.85288	-4.52320	-2.52780
H	2.26906	-3.79431	1.46972
H	0.12356	-4.71860	0.80220
H	5.76664	-4.34847	-1.58345
H	7.78739	-3.03924	-1.08152
H	5.59189	-0.79314	1.92467
H	3.57502	-2.10406	1.43442
H	8.80237	-0.98129	-1.30863
H	10.83051	0.33470	-0.83882
H	9.71863	0.56212	3.35930
H	7.67408	-0.78897	2.88747
H	12.88990	0.67744	0.21985
H	12.03074	2.22241	-0.11639
H	14.01244	1.74274	2.21039
H	13.16147	3.27371	1.88020
H	14.30541	3.42170	-0.38342
H	15.14786	1.89025	-0.06245
H	16.27739	2.93291	1.94970
H	15.43673	4.46368	1.63037
H	17.45050	3.09509	-0.30931
H	17.78674	4.53729	0.70075
H	16.59763	4.63430	-0.63734
O	-3.81422	-0.02012	0.03095
H	-3.09458	-0.54920	0.46397
H	-3.77190	2.03854	-0.03636
H	-5.40949	5.51954	-0.94802
O	-4.84696	4.31968	0.67104
H	-4.78580	5.04349	1.32626
C	-2.37908	3.04300	-3.07142
H	-2.77882	2.71753	-4.05297
O	-0.41580	-4.32113	2.98655
H	-0.65955	-5.20832	3.31985
H	-1.13322	-2.42270	2.97161
C	0.53401	-0.44725	-1.03855

H	0.37885	0.64947	-0.98023
H	0.13595	-0.87989	-0.09329
C	2.00454	-0.79704	-1.12594
H	2.16139	-1.89151	-1.10326
H	2.51982	-0.35561	-0.25210
C	2.51276	-1.25341	-3.52414
H	2.92627	-2.23757	-3.23996
H	1.42639	-1.33557	-3.69264
H	3.03068	-0.86484	-4.41920
C	4.21585	-0.23794	-2.06953
H	4.54459	-1.23284	-1.71869
H	4.76222	0.03882	-2.98874
H	4.39235	0.51394	-1.27990
C	2.25962	1.08070	-2.78183
H	1.20922	1.00962	-3.11561
H	2.34882	1.76512	-1.91955
H	2.89319	1.43893	-3.61261
N	2.73822	-0.29117	-2.37867

Sum of electronic and zero-point Energies= -4164.574553
Sum of electronic and thermal Energies= -4164.486585
Sum of electronic and thermal Enthalpies= -4164.485640
Sum of electronic and thermal Free Energies= -4164.701010

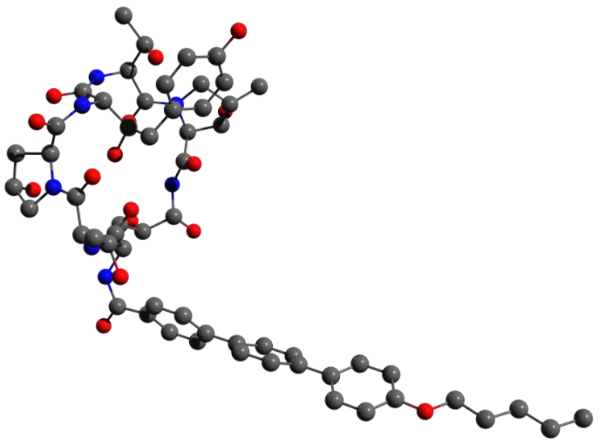

Compound 1:

O	0.41734	-0.16639	4.57095
C	1.26573	-0.34807	3.44358
N	2.32347	0.64262	3.42538
C	1.86163	-1.76730	3.60464
C	2.42376	1.70152	2.56504
O	1.74039	1.83199	1.54257
O	2.86139	-1.76441	4.62204
C	2.46983	-2.26024	2.28933
C	1.38512	-2.92615	1.40784
N	1.17525	-4.32169	1.78383
C	1.66458	-2.69865	-0.08286
O	1.76760	-1.53313	-0.51082
N	1.77700	-3.78595	-0.87196
C	2.02685	-3.68534	-2.31116
C	3.23424	-2.72761	-2.56994
C	0.75842	-3.21504	-3.07979
C	0.89277	-3.43547	-4.58617

N	4.40441	-3.08230	-1.97421
O	3.10281	-1.70243	-3.25327
C	5.55716	-2.15430	-1.81336
C	6.72067	-3.12932	-1.60307
C	6.07856	-4.21257	-0.72608
C	4.70643	-4.40154	-1.37955
O	5.83801	-3.74728	0.60841
N	4.33107	3.26944	2.04718
C	4.00479	4.62261	1.55778
C	2.58735	4.87572	2.10030
C	2.58582	4.09268	3.43468
C	5.36042	2.41671	1.75155
O	3.18975	4.79314	4.51532
C	6.58430	2.83708	0.86667
O	5.41154	1.30149	2.28954
C	5.78135	-1.20875	-2.99832
N	5.67670	0.14572	-2.78105
O	6.09042	-1.64993	-4.11361
C	5.59678	0.95132	-1.56283
C	6.52477	4.04942	-0.09177
N	7.20097	1.63693	0.29180
C	6.83933	0.71863	-0.66677
O	7.56715	-0.26841	-0.84089
C	4.24318	0.91402	-0.79053
C	3.02816	1.16117	-1.71536
C	2.94120	2.58929	-2.19690
C	3.54731	3.01796	-3.39465
C	3.56321	4.37585	-3.76215
C	2.95047	5.33996	-2.93018
C	2.31506	4.92018	-1.73948
C	2.31984	3.56503	-1.38522
O	2.93021	6.67411	-3.22465
C	7.85501	4.27148	-0.81733
H	5.37157	-1.57803	-0.89011
C	2.20355	6.34846	2.21338
C	-2.32500	-4.16143	2.80427
C	-3.56826	-3.60815	2.47724
C	-3.84004	-3.13638	1.16616
C	-2.80921	-3.24661	0.19979
C	-1.55520	-3.77995	0.52823
C	-1.29552	-4.23562	1.84026
C	-5.91894	-1.82730	1.77225
C	-7.17127	-1.28913	1.45039
C	-7.73509	-1.44978	0.15922
C	-6.97524	-2.17451	-0.79395
C	-5.72322	-2.71394	-0.47246
C	-5.16366	-2.55658	0.82010
C	-9.53628	0.31183	0.40549
C	-10.79911	0.84650	0.10107
C	-11.64366	0.17490	-0.81327
C	-11.19173	-1.02745	-1.40999
C	-9.93170	-1.54359	-1.09775
C	-9.06867	-0.88958	-0.17811
O	-12.88500	0.59374	-1.17787
C	-0.00326	-4.89985	2.22995
O	0.01290	-5.93478	2.90537
C	-13.42462	1.78882	-0.59135
C	-14.82767	1.99166	-1.14934
C	-15.51247	3.24533	-0.58764
C	-16.93131	3.45920	-1.13657
C	-17.61561	4.71053	-0.57270
H	0.08244	0.75185	4.54814

H	0.71691	-0.24999	2.47802
H	3.03218	0.53547	4.15796
H	1.01463	-2.43509	3.89491
H	2.40414	-1.53559	5.45729
H	3.27758	-2.99097	2.49997
H	2.94311	-1.41189	1.75471
H	0.42717	-2.38502	1.56349
H	2.00781	-4.83508	2.09799
H	1.57668	-4.69673	-0.44737
H	2.29369	-4.70787	-2.65432
H	1.75550	-2.86908	-4.98659
H	-0.02914	-3.09593	-5.09917
H	1.04124	-4.51395	-4.81321
H	7.58802	-2.63203	-1.13178
H	7.03109	-3.55507	-2.58134
H	6.65905	-5.16436	-0.72162
H	3.96583	-4.69412	-0.61176
H	4.73804	-5.18704	-2.16485
H	6.68779	-3.42005	0.96259
H	4.03864	4.65479	0.45360
H	4.72239	5.37419	1.96140
H	1.87634	4.34384	1.43427
H	1.55748	3.85134	3.77344
H	4.10301	5.02986	4.25675
H	7.30988	3.16033	1.64442
H	5.92149	0.67234	-3.62452
H	5.66036	2.00661	-1.89572
H	8.01783	1.29481	0.80519
H	3.06315	0.42984	-2.54706
H	4.03259	2.27849	-4.05220
H	4.04810	4.69250	-4.70059
H	1.83649	5.67686	-1.09833
H	1.86312	3.24030	-0.43586
H	3.39696	6.82357	-4.07076
H	8.68546	4.42141	-0.09736
H	7.78824	5.17731	-1.45442
H	8.09912	3.40271	-1.46027
H	2.85370	6.87120	2.94436
H	1.15349	6.45812	2.55359
H	2.30093	6.86069	1.23405
H	-2.13765	-4.54877	3.81766
H	-4.35617	-3.56993	3.24522
H	-2.97454	-2.89524	-0.83032
H	-0.80023	-3.85678	-0.27008
H	-5.51339	-1.66405	2.78302
H	-7.73333	-0.74738	2.22711
H	-7.36538	-2.30807	-1.81504
H	-5.17683	-3.28605	-1.23850
H	-8.88852	0.86690	1.10227
H	-11.10947	1.78882	0.57328
H	-11.86119	-1.54751	-2.11220
H	-9.62103	-2.49476	-1.55768
H	-13.45176	1.68881	0.51938
H	-12.77251	2.66086	-0.83392
H	-15.43533	1.08872	-0.91885
H	-14.76447	2.05472	-2.25824
H	-14.89146	4.14265	-0.81398
H	-15.55469	3.17956	0.52412
H	-17.54740	2.55902	-0.91160
H	-16.88571	3.52305	-2.24760
H	-17.70376	4.65609	0.53386
H	-18.63783	4.84105	-0.98500

H	-17.03643	5.62817	-0.81295
O	4.09399	-0.27969	-0.04028
H	3.22000	-0.69116	-0.27069
H	4.28066	1.77348	-0.08813
H	6.32580	4.94325	0.54386
O	5.45362	3.84623	-1.00862
H	5.39231	4.59371	-1.63611
C	3.37745	2.80634	3.06766
H	3.92673	2.44378	3.95990
O	-0.41526	-3.84089	-2.55538
H	-0.38118	-4.79233	-2.78238
H	0.63748	-2.13450	-2.86282
H	2.13591	0.92866	-1.09936

Sum of electronic and zero-point Energies= -3837.385599
Sum of electronic and thermal Energies= -3837.305530
Sum of electronic and thermal Enthalpies= -3837.304585
Sum of electronic and thermal Free Energies= -3837.507506

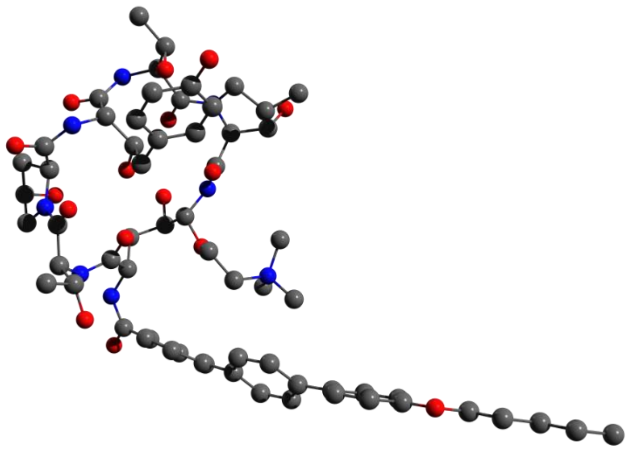

Compound 2:

O	-0.17345	-1.05787	-2.15433
C	-1.50648	-0.55844	-2.30542
N	-1.56505	0.73386	-2.97647
C	-2.26256	-1.63987	-3.12518
C	-1.57547	1.89105	-2.24821
O	-1.19023	1.95043	-1.07205
O	-3.25089	-0.98937	-3.92155
C	-2.93510	-2.69250	-2.22123
C	-1.97151	-3.45729	-1.26097
N	-1.98441	-4.90206	-1.48711
C	-2.28588	-3.05168	0.18574
O	-2.15915	-1.86160	0.54192
N	-2.73478	-4.01446	1.01420
C	-3.14958	-3.73873	2.39039
C	-4.08845	-2.49050	2.42080
C	-1.92000	-3.55034	3.32579
C	-2.30901	-3.61256	4.80198
N	-5.20463	-2.56651	1.64668
O	-3.80028	-1.49127	3.09513
C	-6.03490	-1.38040	1.29589
C	-7.34995	-2.03288	0.84883

C	-6.85722	-3.28109	0.10818
C	-5.75063	-3.79468	1.03053
O	-6.23050	-2.95307	-1.13868
N	-3.06066	3.85668	-2.16242
C	-2.52572	5.08497	-1.54222
C	-1.00785	5.00345	-1.79830
C	-0.91715	4.21711	-3.12826
C	-4.25407	3.20287	-2.01321
O	-1.10215	5.01939	-4.28827
C	-5.49167	3.83498	-1.28850
O	-4.42388	2.10694	-2.56655
C	-6.23379	-0.38516	2.44339
N	-5.80724	0.91107	2.26800
O	-6.80521	-0.72775	3.48757
C	-5.32002	1.66393	1.10985
C	-5.32042	4.94821	-0.23106
N	-6.42086	2.76570	-0.90741
C	-6.40033	1.73186	0.00261
O	-7.30951	0.89219	-0.04200
C	-3.89883	1.29807	0.58208
C	-2.84436	1.25105	1.71158
C	-2.59895	2.59888	2.35126
C	-3.29399	3.01231	3.50626
C	-3.14451	4.30992	4.02846
C	-2.27048	5.22680	3.40272
C	-1.55129	4.82015	2.25571
C	-1.72273	3.52748	1.74396
O	-2.07592	6.50025	3.85797
C	-6.65961	5.40034	0.35668
H	-5.55232	-0.88134	0.43765
C	-0.29064	6.35077	-1.79586
C	1.54530	-5.16089	-2.12855
C	2.78945	-4.65611	-1.73015
C	2.99756	-4.15683	-0.41662
C	1.90511	-4.22220	0.48607
C	0.64912	-4.69371	0.08262
C	0.45049	-5.15075	-1.23917
C	5.47628	-3.72953	-0.76250
C	6.64602	-3.01259	-0.48265
C	6.68327	-2.02431	0.53398
C	5.49249	-1.81294	1.27371
C	4.32381	-2.53104	0.99503
C	4.27665	-3.49757	-0.04229
C	8.80537	-0.88659	-0.23226
C	9.96897	-0.13847	0.00919
C	10.27456	0.27864	1.32590
C	9.39225	-0.06633	2.37919
C	8.23653	-0.80696	2.12068
C	7.91253	-1.23974	0.80716
O	11.37115	1.00075	1.67699
C	-0.87305	-5.69484	-1.71280
O	-0.96274	-6.79570	-2.26523
C	12.32417	1.37106	0.66739
C	13.44651	2.14655	1.34575
C	14.53366	2.59701	0.36029
C	15.67272	3.38019	1.03098
C	16.75933	3.82885	0.04614
H	-1.96287	-0.43229	-1.30510
H	-2.09963	0.72043	-3.85467
H	-1.49581	-2.13227	-3.76997
H	-3.52258	-1.60736	-4.62649
H	-3.43212	-3.43182	-2.88251

H	-3.74924	-2.19381	-1.65671
H	-0.93865	-3.09604	-1.44113
H	-2.85636	-5.31287	-1.84203
H	-2.70232	-4.98015	0.67337
H	-3.72313	-4.62915	2.72745
H	-3.04176	-2.81891	5.04380
H	-1.40910	-3.47592	5.43433
H	-2.76125	-4.59834	5.04721
H	-7.94851	-1.34843	0.22111
H	-7.94491	-2.32423	1.74076
H	-7.65706	-4.04468	-0.03254
H	-4.99190	-4.33885	0.43920
H	-6.15754	-4.47622	1.80827
H	-6.85163	-2.39529	-1.64627
H	-2.75381	5.10158	-0.46099
H	-2.96414	5.99166	-2.01952
H	-0.57518	4.34155	-1.01962
H	0.07375	3.73458	-3.25441
H	-1.97240	5.46108	-4.22067
H	-6.00791	4.34896	-2.12812
H	-6.06876	1.49274	3.06934
H	-5.18141	2.70322	1.46933
H	-7.17526	2.60988	-1.58194
H	-3.16155	0.49057	2.45268
H	-3.97872	2.30893	4.00711
H	-3.70191	4.61516	4.92987
H	-0.87420	5.53996	1.76957
H	-1.20128	3.22756	0.82244
H	-2.63074	6.64514	4.64994
H	-7.34616	5.75559	-0.43881
H	-6.49524	6.23808	1.06526
H	-7.15073	4.56814	0.89885
H	-0.65645	6.99555	-2.62086
H	0.80226	6.21583	-1.92948
H	-0.45448	6.88476	-0.83730
H	1.40504	-5.53661	-3.15375
H	3.60377	-4.61872	-2.46915
H	2.02366	-3.89437	1.52955
H	-0.16387	-4.71106	0.82618
H	5.50578	-4.49726	-1.55022
H	7.55973	-3.24175	-1.05243
H	5.46575	-1.03816	2.05536
H	3.41555	-2.29446	1.56920
H	8.57628	-1.17782	-1.26953
H	10.62271	0.12179	-0.83458
H	9.64583	0.25430	3.40119
H	7.58283	-1.08083	2.96363
H	12.72136	0.45729	0.16583
H	11.82883	1.99468	-0.11360
H	13.89135	1.50676	2.13972
H	13.00771	3.02946	1.86092
H	14.07606	3.22566	-0.43809
H	14.95675	1.70726	-0.16061
H	16.12513	2.75039	1.83040
H	15.24625	4.26757	1.55177
H	17.22897	2.95745	-0.45900
H	17.56591	4.39674	0.55491
H	16.33709	4.48234	-0.74757
O	-3.89166	0.10254	-0.18882
H	-3.30362	-0.56869	0.24242
H	-3.60826	2.13299	-0.08898
H	-4.86532	5.81714	-0.76123

O	-4.43492	4.46591	0.77518
H	-4.31091	5.13539	1.47750
C	-2.04400	3.16169	-2.97956
H	-2.45437	2.90653	-3.97721
O	-0.89512	-4.49553	3.00797
H	-1.20808	-5.38377	3.27414
H	-1.48814	-2.55725	3.08766
C	0.49670	-0.62748	-0.96228
H	0.30063	0.44858	-0.77695
H	0.10655	-1.17989	-0.07680
C	1.97445	-0.93705	-1.06237
H	2.15281	-2.02484	-1.13907
H	2.46849	-0.57112	-0.14250
C	2.52488	-1.16860	-3.47991
H	2.95024	-2.16810	-3.27719
H	1.44241	-1.25085	-3.67264
H	3.05169	-0.69496	-4.32770
C	4.18929	-0.27208	-1.91014
H	4.51610	-1.28983	-1.62831
H	4.75013	0.07346	-2.79682
H	4.34662	0.42027	-1.06425
C	2.22445	1.08638	-2.53082
H	1.18429	1.02741	-2.89803
H	2.27555	1.68258	-1.60260
H	2.87123	1.53781	-3.30395
N	2.71806	-0.31010	-2.24925
H	-1.91094	0.89308	1.23077

Sum of electronic and zero-point Energies= -4089.404203
Sum of electronic and thermal Energies= -4089.317020
Sum of electronic and thermal Enthalpies= -4089.316076
Sum of electronic and thermal Free Energies= -4089.530985

4. References

(1) Garcia-Effron, G.; Park, S.; Perlin, D. S. Correlating Echinocandin MIC and Kinetic Inhibition of Fks1 Mutant Glucan Synthases for Candida Albicans: Implications for Interpretive Breakpoints. *Antimicrob. Agents Chemother.* **2009**, *53* (1), 112–122. https://doi.org/10.1128/AAC.01162-08.

(2) Ben-Ami, R.; Garcia-Effron, G.; Lewis, R. E.; Gamarra, S.; Leventakos, K.; Perlin, D. S.; Kontoyiannis, D. P. Fitness and Virulence Costs of Candida Albicans FKS1 Hot Spot Mutations Associated With Echinocandin Resistance. *J. Infect. Dis.* **2011**, *204* (4), 626–635. https://doi.org/10.1093/infdis/jir351.

(3) Carreté, L.; Ksiezopolska, E.; Pegueroles, C.; Gómez-Molero, E.; Saus, E.; Iraola-Guzmán, S.; Loska, D.; Bader, O.; Fairhead, C.; Gabaldón, T. Patterns of Genomic Variation in the Opportunistic Pathogen Candida Glabrata Suggest the Existence of Mating and a Secondary Association with Humans. *Curr. Biol.* **2018**, *28* (1), 15-27.e7. https://doi.org/10.1016/J.CUB.2017.11.027.

(4) Ksiezopolska, E.; Schikora-Tamarit, M. À.; Beyer, R.; Nunez-Rodriguez, J. C.; Schüller, C.; Gabaldón, T. Narrow Mutational Signatures Drive Acquisition of Multidrug Resistance in the Fungal Pathogen Candida Glabrata. *Curr. Biol.* **2021**, *31* (23), 5314-5326.e10. https://doi.org/10.1016/j.cub.2021.09.084.

(5) Gaussian 09, Revision D.01, M. J. Frisch, G. W. Trucks, H. B. Schlegel, G. E. Scuseria, M. A. Robb, J. R. Cheeseman, G. Scalmani, V. Barone, B. Mennucci, G. A. Petersson, H. Nakatsuji, M. Caricato, X. Li, H. P. Hratchian, A. F. Izmaylov, J. Bloino, G. Zheng, J. L. Sonnengerg, M. Hada, M. Ehara, K. Toyota, R. Fukuda, J. Hasegawa, M. Ishida, T. Nakajima, Y. Honda, O. Kitao, H. Nakai, T. Vreven, J. A. Montgomery Jr, J. E. Peralta, F. Ogliaro, M. Bearpark, J. J. Heyd, E. Brothers, K. N. Kudin, V. N. Staroverov, T. Keith, R. Kobayashi, J. Normand, K. Raghavachari, A. Rendell, J. C. Burant, S. S. Iyengar, J. Tomasi, M. Cossi, N. Rega, N. J. Millam, M. Klene, J. E. Knox, J. B. Cross, V. Bakken, C. Adamo, J. Jaramillo, R. Gomperts, R. E. Stratmann, O. Yazyev, A. J. Austin, R. Cammi, C. Pomelli, J. W. Ochterski, R. L. Martin, K. Morokuma, V. G. Zakrzewski, G. A. Voth, P. Salvador, J. J. Dannenberg, S. Dapprich, A. D. Daniels, Ö. Farkas, J. B. Foresman, J. V. Ortiz, J. Cioslowski and D. J. Fox, Gaussian, Inc., Wallingford CT, 2010.

(6) Becke, A. D. Density-Functional Exchange-Energy Approximation with Correct Asymptotic Behavior. *Phys. Rev. A* **1988**, *38* (6), 3098. https://doi.org/10.1103/PhysRevA.38.3098.

(7) Perdew, J. P. Density-Functional Approximation for the Correlation Energy of the Inhomogeneous Electron Gas. *Phys. Rev. B* **1986**, *33* (12), 8822. https://doi.org/10.1103/PhysRevB.33.8822.

(8) Weigend, F.; Ahlrichs, R. Balanced Basis Sets of Split Valence, Triple Zeta Valence and Quadruple Zeta Valence Quality for H to Rn: Design and Assessment of Accuracy. *Phys. Chem. Chem. Phys.* **2005**, *7* (18), 3297–3305. https://doi.org/10.1039/B508541A.

(9) Barone, V.; Cossi, M. Quantum Calculation of Molecular Energies and Energy Gradients in Solution by a Conductor Solvent Model. *J. Phys. Chem. A* **1998**, *102* (11), 1995–2001. https://doi.org/10.1021/JP9716997.

(10) Cossi, M.; Rega, N.; Scalmani, G.; Barone, V. Energies, Structures, and Electronic Properties of Molecules in Solution with the C-PCM Solvation Model. *J. Comput. Chem.* **2003**, *24* (6), 669–681. https://doi.org/10.1002/JCC.10189.
